# Repurposing a plant peptide cyclase for targeted lysine acylation

**DOI:** 10.1038/s41557-024-01520-1

**Published:** 2024-05-24

**Authors:** Fabian B. H. Rehm, Tristan J. Tyler, Yan Zhou, Yen-Hua Huang, Conan K. Wang, Nicole Lawrence, David J. Craik, Thomas Durek

**Affiliations:** Institute for Molecular Bioscience, Australian Research Council Centre of Excellence for Innovations in Peptide and Protein Science, The University of Queensland, Brisbane, Queensland Australia

**Keywords:** Ligases, Peptides

## Abstract

Transpeptidases are powerful tools for protein engineering but are largely restricted to acting at protein backbone termini. Alternative enzymatic approaches for internal protein labelling require bulky recognition motifs or non-proteinogenic reaction partners, potentially restricting which proteins can be modified or the types of modification that can be installed. Here we report a strategy for labelling lysine side chain ε-amines by repurposing an engineered asparaginyl ligase, which naturally catalyses peptide head-to-tail cyclization, for versatile isopeptide ligations that are compatible with peptidic substrates. We find that internal lysines with an adjacent leucine residue mimic the conventional N-terminal glycine–leucine substrate. This dipeptide motif enables efficient intra- or intermolecular ligation through internal lysine side chains, minimally leaving an asparagine C-terminally linked to the lysine side chain via an isopeptide bond. The versatility of this approach is demonstrated by the chemoenzymatic synthesis of peptides with non-native C terminus-to-side chain topology and the conjugation of chemically modified peptides to recombinant proteins.

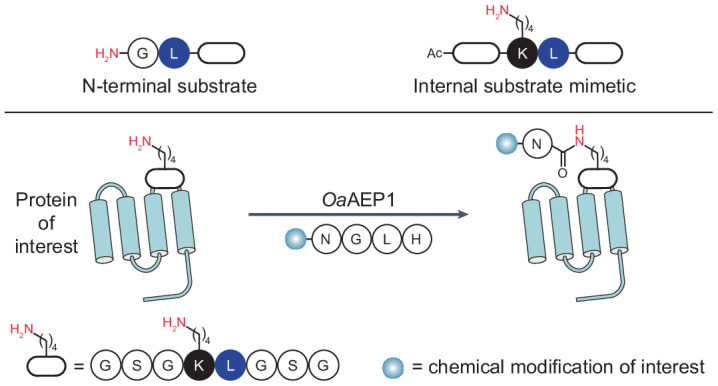

## Abstract (web summary)

Plant asparaginyl endopeptidases that function preferentially as transpeptidases naturally catalyse the head-to-tail cyclization of plant peptides. Using substrate mimicry and reaction optimization, their function has now been repurposed to catalyse intermolecular isopeptide bond formation on diverse peptide and protein substrates.

## Main

The attachment of defined chemical moieties of interest to a specific site on a protein or peptide is an invaluable strategy to study protein/peptide function or to develop new therapeutics and medical imaging agents^1^. Despite ongoing advances in chemical strategies for this purpose^2^, important challenges persist. For example, the modification of cysteine thiols, a widely used approach in chemical protein modification, suffers from competing reactions with other cysteine residues or disulfides in the protein. Using chemical approaches, regioselective protein modification is rarely achieved and, when it is, it is only after substantial case-by-case reaction optimization.

**Extended Data Fig. 1:**
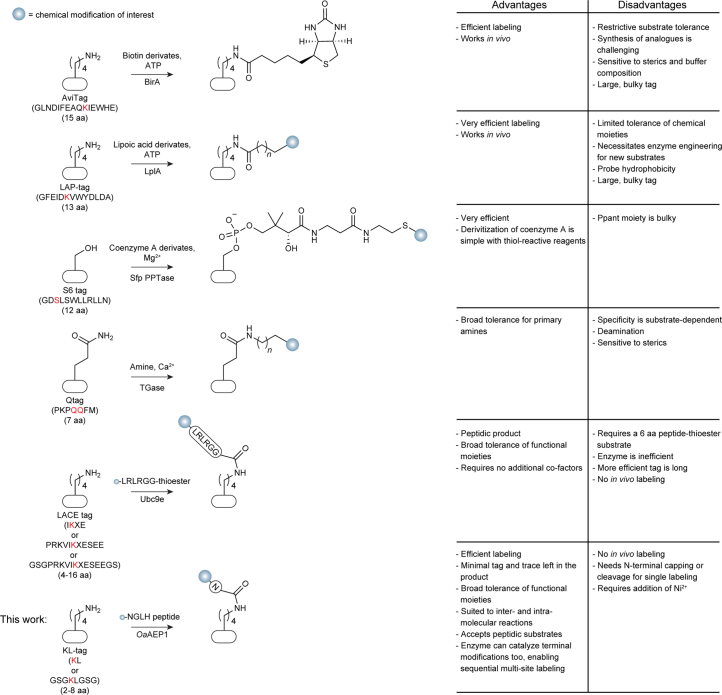
Overview of strategies for labelling protein side chains. Existing chemoenzymatic methods and the approach described in this Article are summarised. Recognition sequences as well as advantages and disadvantages are listed for each approach.

Enzymatic methods for protein and peptide modification have transformed our ability to generate protein/peptide bioconjugates under mild reaction conditions^3^. A range of established tools for protein modification at their C or N termini are now available, including inteins^4^, transpeptidases^5^ and engineered proteases^6^, whereas approaches for internal protein modifications are more limited. Existing methodologies largely rely on ligases or transferases^7^, such as biotin ligase, lipoic acid ligase or transglutaminase, which are capable of transferring derivatives of their cognate metabolites to amino acid (aa) side chains within a specific recognition motif (7–15 aa; Extended Data Fig. 1). However, the resultant linkages are often bulky and artificial, substrate synthesis can be complex, and engineering new enzyme variants with specificities suitable to a new modification of interest is a considerable hurdle to overcome. An alternative strategy is to utilize engineered, split bacterial adhesins, such as SpyTag/SpyCatcher^8^ or DogTag/DogCatcher^9^, which rapidly and efficiently ligate via spontaneous transamidation but leave a bulky adhesin domain (~14 kDa) in the ligated product.

Another noteworthy technique relies on the use of the E2 SUMO-conjugating enzyme Ubc9, which recognizes a 6-aa C-terminal-thioester motif and ligates it to a Lys residue embedded in a 4-aa peptide tag, although in practice longer tags of 11 aa (sometimes with additional flanking GSG or GS linkers, 16 aa in total) were used^10^. This approach necessitates preparation of C-terminal thioester substrates, leaves at least 6 aa connecting the Lys to the attached constituent, requires 4 molar equivalents of enzyme relative to the limiting substrate, and reactions proceed slowly (up to 24 h at 30 °C). In follow-up work, the authors have shown that an E1 enzyme can be used together with Ubc9 to overcome the requirement for a C-terminal thioester substrate. However, this approach only enables the attachment of ubiquitin to proteins, effectively reconstituting the natural ubiquitin conjugating system^11^.

In recent work by Piel and colleagues, an enzymatic splicing strategy was employed to enable the generation of dienophiles for subsequent labelling with tetrazine reagents^12^. This approach utilizes the splicease complex PlpXY to form an α-keto-β-amino acid via tyramine excision at a Tyr–Gly motif within an 11–24-aa tag sequence. However, because the tetrazine cycloaddition directly modifies the polypeptide backbone and the tag sequence contains multiple prolines, the selection of appropriate sites for tag insertion is challenging and may simply not be tolerated by certain proteins.

In this Article we demonstrate that the asparaginyl ligase *Oa*AEP1, which naturally catalyses intramolecular transpeptidation reactions to yield cyclic peptides in the plant *Oldenlandia affinis*^13,14^, can be repurposed to catalyse targeted intra- or intermolecular Lys acylation. Existing enzymatic approaches utilize enzymes in reactions that are highly analogous to their natural functions. By contrast, our approach constitutes a paradigm shift—we repurpose *Oa*AEP1 from peptide to isopeptide bond formation and from acting at terminal to internal sites. Despite a range of previous developments and applications^15–28^, asparaginyl ligases have thus far been restricted to terminal peptide and protein modification. The only described means of labelling at internal sites with an asparaginyl ligase utilized an expanded genetic code to encode a synthetic Lys derivative bearing a dipeptide on the Lys side chain, effectively providing a ‘second N terminus’ for labelling^29^. Our enzymatic Lys acylation strategy is compatible with peptidic substrates, minimally leaves an Asn C-terminally isopeptide-linked directly to a Lys residue with an adjacent Leu and requires only catalytic quantities of enzyme relative to the limiting substrate. *Oa*AEP1-catalysed Lys acylation is complementary and largely orthogonal to existing enzymatic strategies and provides great utility for peptide engineering applications where existing approaches are not suitable due to their bulky recognition sites and linkages. We demonstrate a chemoenzymatic strategy for accessing peptides with unusual C terminus-to-side chain topology, previously only accessible via chemical synthesis, and a straightforward approach for generating branched dendrimer-like peptide structures. Furthermore, we demonstrate targeted Lys labelling in multiple protein substrates, including a single-domain antibody heterodimer, and show that *Oa*AEP1 can be used in distinct, sequential steps to modify defined internal and terminal sites in a protein substrate.

## Results and discussion

### Enzymatic Lys side chain acylation by a peptide cyclase

**Fig. 1:**
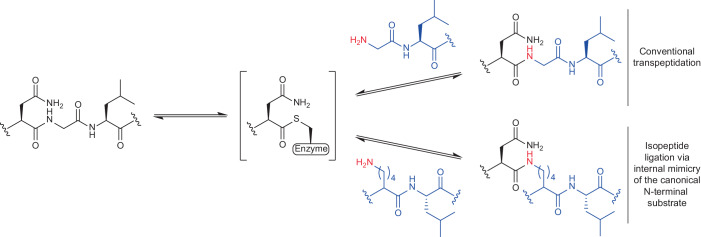
Overview of conventional transpeptidation and non-canonical isopeptide ligation reactions catalysed by the asparaginyl ligase *Oa*AEP1. The reactions proceed via a thioester-linked acyl-enzyme intermediate, as shown.

The Cys247Ala mutant of *Oa*AEP1, a catalytically improved asparaginyl ligase, acts on Asn–Gly–Leu (P1–P1′–P2′) sequences and, via a thioester-linked acyl-enzyme intermediate, transfers the P1 Asn α-carboxyl to a compatible N-terminal acceptor nucleophile (such as the α-amine of a Gly–Leu–peptide, P1′′–P2′′), where hydrophobic, aliphatic residues are preferred in the P2′′ position (Fig. 1)^14^. The natural function of the enzyme is to catalyse this reaction in the context of the head-to-tail (C terminus-to-N terminus) cyclization of cyclotides, disulfide-knotted peptides found in plants^13,15,18,30^.

**Fig. 2:**
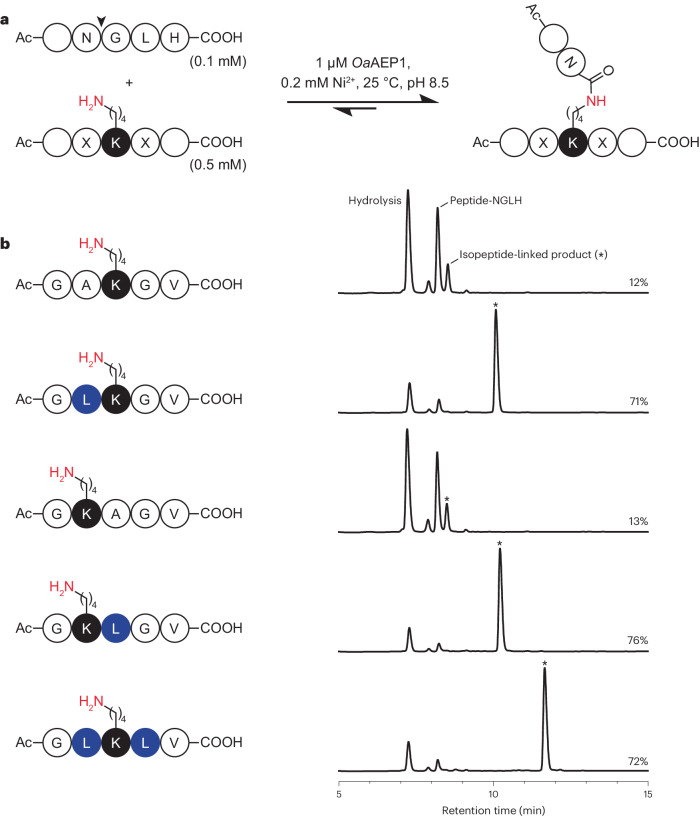
Targeted Lys acylation by an asparaginyl ligase. **a**, Scheme of *Oa*AEP1-catalysed isopeptide ligation (X indicates the positions adjacent to the Lys residue that were varied; the scissile bond in the NGL sequence is indicated with an arrow). **b**, RP-HPLC (280 nm; 15 min 20–50% acetonitrile gradient) analysis of the ligation of a model NGLH-containing acyl donor peptide (Ac-RWRGWRNGLH, 0.1 mM) to Lys-containing acyl acceptor peptides (0.5 mM) as catalysed by 1 µM *Oa*AEP1 in HEPES buffer containing 0.2 mM NiSO_4_ (pH 8.5) after 1.5 h at 25 °C (*n* = 3 independent experiments with similar results; representative data shown). Relative peak areas were used to calculate conversion of the limiting Ac-RWRGWRNGLH peptide to the isopeptide-linked product. The hydrolysis product is Ac-RWRGWRN-OH. We note that peaks for the Lys-containing acyl acceptor peptides are not detected at 280 nm. See Supplementary Fig. 1 for MALDI–TOF MS spectra of these reactions and Supplementary Figs. 2 and 3 for RP-HPLC and MALDI–TOF MS analysis of equivalent reactions without NiSO_4_.

Notably, the P1′–P2′ leaving group released from the recognition sequence can compete with the desired incoming N-terminal nucleophile, resulting in reaction reversal. To suppress this potential equilibrium, the Asn–Gly–Leu sequence can be extended with a His residue to instead release Gly–Leu–His, which can be quenched via metal complex formation upon addition of Ni^2+^ to the reaction mixture^22^. As desired incoming N-terminal nucleophile sequences must no longer out-compete optimal leaving group sequences (P1′–P2′; H_2_N–Gly–Leu), these improved reactions are effectively more promiscuous and allow less optimal mimetics of the canonical Gly–Leu to be efficiently incorporated^19^. To test whether this promiscuity could extend beyond standard terminal peptide/protein modifications, which have been exclusively examined thus far, we set out to investigate whether internal Lys residues could be acylated by *Oa*AEP1. To this end, we synthesized a model acyl donor peptide (Ac-RWRGWRNGLH) and a series of short acceptors peptides bearing an internal Lys residue with an ε-amine as the sole amine nucleophile (Fig. 2). All peptides were N-terminally acetylated to simplify analysis of the reactions by eliminating the possibility of ligation through the α-amine. With the aim of mimicking the canonical N-terminal Gly–Leu nucleophile, our series of Lys-containing peptides included variants with a Leu residue at either or both Lys-adjacent positions (Fig. 2).

Analysis of *Oa*AEP1-catalysed reactions (1 µM enzyme) between the sets of peptides via reverse-phase (RP) HPLC and matrix-assisted laser desorption ionization time of flight mass spectrometry (MALDI-TOF MS; Fig. 2 and Supplementary Fig. 1) indeed revealed that the presence of a Leu at either or both adjacent positions enabled efficient (>70% product, relative to the limiting substrate) isopeptide ligations, using only 0.5 mM of the Lys-containing acyl acceptor peptide and 0.1 mM Ac-RNGLH, in a Ni^2+^-dependent manner (Supplementary Figs. 2 and 3). These reactions were run at slightly basic pH (8.5) as this has been shown to favour transpeptidation over hydrolysis reaction outcomes^21,31,32^. Reactions where the Lys–Leu-containing substrate was supplied in just twofold excess (0.2 mM), which is comparable to standard C-to-N-terminal transpeptidation conditions^14,17^, yielded >60% isopeptide-linked product (Supplementary Figs. 4 and 5; tenfold excess yielded 85%). Thus, *Oa*AEP1 is able to catalyse targeted intermolecular Lys acylation, minimally leaving an Asn isopeptide linked to a Lys residue with an adjacent Leu in the product. We note that, although a Ni^2+^-mediated quenching approach was employed to enhance the reactions, other comparable strategies for shifting the reaction equilibrium toward product formation are available^33–35^. The use of Ni^2+^ may not be suitable for labelling peptides or proteins that must chelate specific metal ions to function.

**Extended Data Fig. 2:**
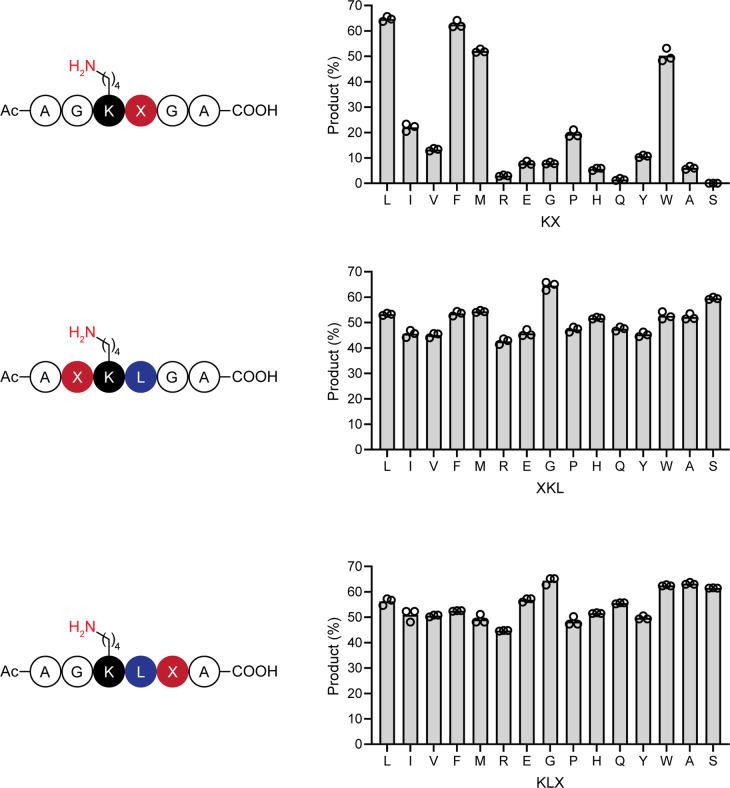
Screen of Lys- or Lys-Leu-adjacent residues for isopeptide labelling with *Oa*AEP1. Reactions containing 100 µM of a model NGLH-containing acyl donor peptide (Ac-RWRGWRNGLH), 200 µM of a Lys-containing acyl acceptor peptide (as indicated in the figure), 200 µM Ni^2+^, and 1 µM *Oa*AEP1 were run in 100 mM HEPES buffer, pH 8.5, for 3 h at 25 °C. Conversion (%) of the NGLH-containing substrate to isopeptide-linked product was determined via RP-HPLC (*n* = 3 independent experiments). Source data

**Extended Data Fig. 3:**
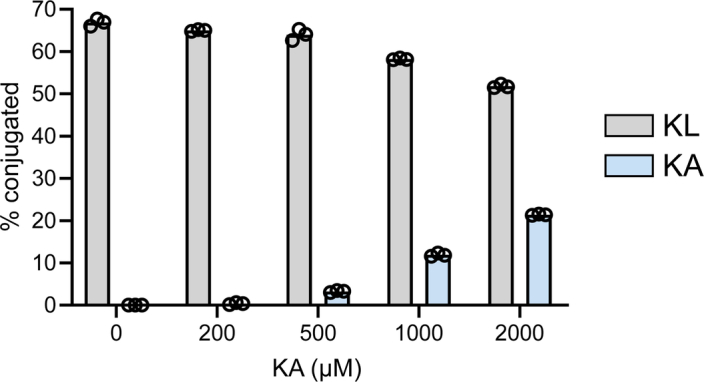
Isopeptide ligation on a Lys-Leu peptide in the presence of increasing Lys-Ala peptide concentrations. Reactions containing 100 µM of a model NGLH-containing acyl donor peptide (Ac-RWRGWRNGLH), 200 µM of a Lys-Leu-containing peptide, 0-2,000 µM of a Lys-Ala-containing peptide (as indicated), 200 µM Ni^2+^, and 1 µM *Oa*AEP1 were run in 100 mM HEPES buffer, pH 8.5, for 3 h at 25 °C. Conversion (%) of the NGLH-containing substrate to one or the other isopeptide-linked products was determined via RP-HPLC (*n* = 3 independent experiments). Source data

To further characterize these isopeptide-forming reactions, we examined a series of peptides where the Lys-adjacent amino acid was varied to a set of amino acids with diverse physicochemical properties (excluding Cys, Lys, Asp, Asn and Thr; Extended Data Fig. 2). This revealed that Lys–Leu-containing peptides are most efficiently incorporated by the enzyme, but other hydrophobic residues (Phe, Met and Trp) are also tolerated. This Lys–Xaa substrate-specificity profile essentially mirrors canonical N-terminal Gly–Xaa substrate specificity and validates internal Lys–Leu sequences as legitimate substrate mimetics^33^. Additional peptide analogues varying the identity of the residues either side of the Lys–Leu sequence revealed a broad amino acid tolerance, although a flanking Gly residue was somewhat preferred (Extended Data Fig. 2). We additionally examined ligation at a Lys–Leu sequence in the presence of a competing Lys–Ala peptide (Extended Data Fig. 3). At equimolar concentrations (0.2 mM), we observed a 130-fold selectivity for Lys–Leu over Lys–Ala. As expected, this ratio diminished with higher Lys–Ala peptide concentrations, but even at tenfold excess (2 mM) relative to Lys–Leu (0.2 mM) we saw a 2.5-fold greater level of ligation at Lys–Leu compared with Lys–Ala.

**Extended Data Fig. 4:**
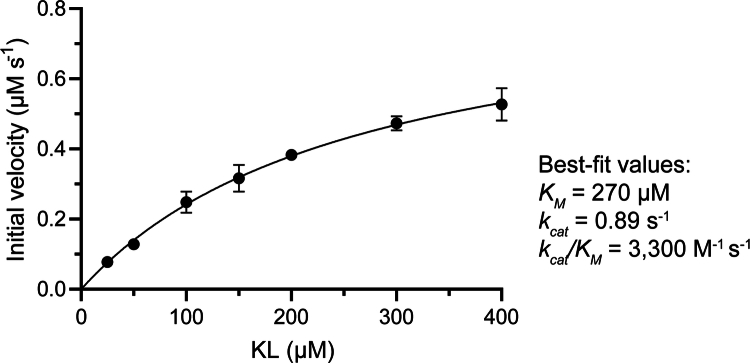
Michaelis-Menten characterisation of *Oa*AEP1-catalysed isopeptide ligation. Initial velocities were determined, via analysis of product formation by RP-HPLC, for reactions containing 1 mM of a model NGLH-containing acyl donor peptide (Ac-RWRGWRNGLH), 0-400 µM of a Lys-Leu-containing acyl acceptor peptide (Ac-AG**KL**GA; concentration as indicated), 2 mM Ni^2+^, and 1 µM *Oa*AEP1 in 100 mM HEPES buffer, pH 8.5, at 25 °C. The mean values of four independent replicates are shown, errors bars indicate the standard deviation. Best-fit values for fitting the Michaelis-Menten model were determined using GraphPad Prism. Source data

Next, we determined the kinetic parameters for a reaction between our model NGLH-containing peptide and an N-terminally acetylated Lys–Leu-containing peptide. We provided the NGLH-containing acyl donor peptide in excess, varied the concentration of the Lys–Leu peptide, and quantified product formation via RP-HPLC (Extended Data Fig. 4). We determined a *K*_M_ of 270 µM and a catalytic efficiency (*k*_cat_/*K*_M_) of 3,300 M^−1^ s^−1^ for this reaction. This catalytic efficiency is approximately tenfold lower than previously determined for *Oa*AEP1-catalysed intramolecular peptide cyclization (at pH 6)^14^, but 15-fold higher than transpeptidation catalysed by wild-type *Staphylococcus aureus* sortase A, an enzyme that has been extensively utilized for protein engineering^36,37^. Thus, the catalytic efficiency of the reaction is sufficient for practical use.

We additionally generated and studied homology models of an *Oa*AEP1 acyl-enzyme intermediate (using Ac-RN as a model acyl donor) in complex with an acyl acceptor peptide substrate, either **GL**GV-NH_2_, Ac-G**KL**GV-NH_2_, **G**AGV-NH_2_ or Ac-G**K**AGV-NH_2_, respectively, using molecular dynamics (Supplementary Fig. 6). This indicated that the peptide substrate can remain transiently bound to the active site, with residence times being longer in some simulations than others, measured according to the distance between the N-terminal α-amine of **GL**GV-NH_2_ or **G**AGV-NH_2_ or the Lys ε-amine of Ac-G**KL**GV-NH_2_ or Ac-G**K**AGV-NH_2_ and the carbonyl carbon electrophile of the thioester-linked Asn. Shorter distances (<5 Å) occurred most frequently for the **GL**GV-NH_2_ and Ac-G**KL**GV-NH_2_ substrates compared to the Ac-G**K**AGV-NH_2_ and **G**AGV-NH_2_ control peptides, in agreement with our experimental characterization (Fig. 2).

### Peptide cyclization via enzymatic isopeptide bond formation

**Fig. 3:**
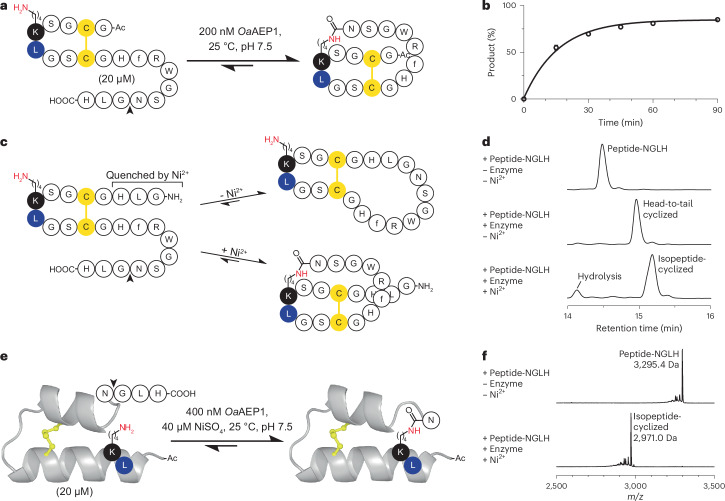
Isopeptide bond cyclization via an asparaginyl ligase. **a**, Scheme of *Oa*AEP1-catalysed isopeptide cyclization of a model peptide substrate (Ac-GCGS**KL**GSCGHfRWGSNGLH). **b**, Isopeptide-cyclized product formation of the model peptide substrate shown in **a**, quantified by RP-HPLC (relative peak integrals; *n* = 3 independent experiments; mean ± s.d.; error bars are too small to see at some points). Representative RP-HPLC chromatograms and MALDI–TOF MS spectra are shown in Supplementary Fig. 7. **c**, Scheme of Ni^2+^-mediated control of the cyclization site, and thus product topology, in a peptide bearing an N-terminal Ni^2+^-quenchable GLH sequence (GLHGCGS**KL**GSCGHfRWGSNGLH). **d**, RP-HPLC (214 nm; 30 min 5–60% acetonitrile gradient) analysis of the reactions with and without 100 µM Ni^2+^ as shown in **c**. Reactions were conducted as in **a** for 3 h. MALDI–TOF MS spectra for these spectra are shown in Supplementary Fig. 11. **e**, Scheme of *Oa*AEP1-catalysed synthesis of an isopeptide-cyclized peptide where the Lys residue was inserted into a helical segment (Ac-GSTT**LK**NIYNTCRFGGGSRTLCARLSGNGLH). **f**, MALDI–TOF MS analysis of the reaction described in **e** after 3 h. Further analyses and reaction optimizations are shown in Supplementary Figs. 12–16. Source data

Having established the feasibility of *Oa*AEP1-catalysed intermolecular isopeptide ligations, we hypothesized that, unlike existing enzymatic approaches for targeted side chain modifications, which necessitate bulky recognition motifs, the minimal sequence requirements of this strategy would be ideally suited to the precision engineering of peptides and small proteins. To test this hypothesis, we synthesized a model peptide bearing a C-terminal recognition sequence and an internal Lys–Leu motif (Ac-GCGS**KL**GSCGHfRWGSNGLH) to explore the possibility of intramolecularly generating unnatural C terminus-to-side chain peptide topologies (Fig. 3a). Notably, we introduced a Cys residue on either side of the internal Lys–Leu motif in this model peptide to allow us to investigate the potential effects of constraining this sequence via a disulfide bond. We added a melanocortin receptor-targeting sequence (HfRW, where ‘f’ is d-Phe) into the loop that would form upon isopeptide cyclization^38,39^. We found that *Oa*AEP1 could rapidly (within 1 h) and efficiently (0.01 equiv. of enzyme) generate C terminus-to-side chain cyclized peptides, either when the peptide was oxidized or reduced (Fig. 3b and Supplementary Figs. 7 and 8). The isopeptide bond between the side chain amine of Lys5 and α-carboxyl of Asn17 was confirmed by NMR spectroscopy, revealing new nuclear Overhauser effect cross peaks between the Lys5 H^ζ^ proton and the Asn17 H^α^ and H^N^ protons upon enzymatic cyclization (Supplementary Fig. 9). The reaction exclusively yielded hydrolysis product when the Lys within the substrate was replaced by Ala (Supplementary Fig. 10).

Given that the nucleophilicity of N-terminal Gly–Leu–His sequences can be quenched by the addition of Ni^2+^, we previously utilized Ni^2+^ addition to suppress Gly–Leu–His-containing substrates such that an alternative substrate could be preferentially labelled^19^. Based on that finding, we synthesized a variant of the model peptide (**GL**HGCGS**KL**GSCGHfRWGSNGLH) that can be head-to-tail cyclized in the absence of Ni^2+^, when the N terminus is unquenched and Lys acylation proceeds inefficiently, but C terminus-to-side chain cyclized in its presence, effectively allowing for simple control of the cyclic peptide topology via Ni^2+^ (Fig. 3c,d and Supplementary Fig. 11).

Having assessed model peptides, we next took inspiration from work by Kent and colleagues on the multi-step total chemical synthesis of a topological derivative of the small protein crambin, which has its C terminus intramolecularly joined to a Lys side chain^40^. We synthesized a linear, truncated crambin-like thionine peptide (Δ-Uf1a), that we had earlier isolated from a New Zealand stinging nettle^41^. We equipped this peptide with an additional C-terminal Asn–Gly–Leu–His recognition motif and a Leu–Lys sequence embedded into a helical segment (Ac-GSTT**LK**NIYNTCRFGGGSRTLCARLSGNGLH, Fig. 3e). Addition of *Oa*AEP1 at 0.02 molar equivalents relative to this substrate enabled its near-quantitative C terminus-to-side chain cyclization in one step (Fig. 3f and Supplementary Figs. 12–15). Analysis of the product by circular dichroism (CD) spectroscopy revealed that the overall helical structure was maintained, albeit with slightly reduced helicity relative to the non-cyclized substrate (Supplementary Fig. 16). Thus, our enzymatic approach provides ready access to peptide topologies previously only accessible by complex multi-step chemical synthesis.

### Chemoenzymatic synthesis of a branched peptide

**Fig. 4:**
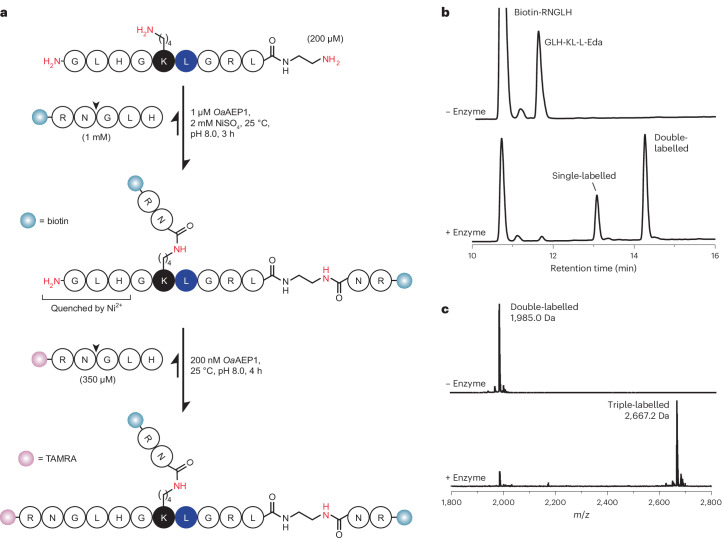
Sequential C-terminal, internal and N-terminal modifications of a linear peptide. **a**, Scheme of the multi-site labelling of a small linear acyl acceptor peptide bearing a Ni^2+^-quenchable N-terminal GLH sequence, an internal KL sequence, and a C-terminal Leu-ethylenediamine (L-Eda) motif that mimics an N-terminal GL substrate (GLHG**KL**GRL-Eda). **b**, RP-HPLC (214 nm; 30 min 5–60% acetonitrile gradient) analysis of the first labelling step with biotin-RNGLH, conducted in the presence of Ni^2+^ as shown in **a**. A high concentration of NiSO_4_ was used as Ni^2+^ functions to quench both the N terminus of the acyl acceptor peptide and the GLH leaving group released from the acyl donor substrate. In the upper chromatogram the biotin-RNGLH peak extends beyond the height of the *y*-axis—the scale for the upper and lower panels is the same. **c**, MALDI–TOF MS analysis of purified C-terminally and internally labelled product generated in **b** and its N-terminal labelling with TAMRA-RNGLH in the absence of Ni^2+^ as shown in **a**. RP-HPLC spectra of this reaction are shown in Supplementary Fig. 17.

With the addition of targeted Lys acylation to the repertoire of site-specific reactions catalysed by *Oa*AEP1, this enzyme is now able to attach NGLH-containing acyl donor substrates to N termini, C termini and Lys side chains^19,22^. To demonstrate this versatility, we synthesized a minimal, linear peptide bearing an N-terminal Ni^2+^-quenchable Gly–Leu–His sequence, an internal Lys–Leu sequence, and a C-terminal Leu–ethylenediamine motif (a C-terminal mimetic of the conventional N-terminal Gly–Leu substrate^19^) (GLHG**KL**GRL-Eda). We then used *Oa*AEP1 to attach biotin-RN to the C terminus and the Lys side chain in the first step (in the presence of Ni^2+^, thus yielding no triple-labelled product) (Fig. 4a,b), then purified the double-labelled product via RP-HPLC, and subsequently labelled the N terminus with a TAMRA-RNGLH peptide to yield a triple-labelled product (Fig. 4a,c). Thus, a single enzyme can be used to generate branching at multiple sites within a short, linear peptide. We foresee that this basic approach could be readily expanded to generate more complex dendrimer-like structures in a highly controlled manner.

### Targeted enzyme-catalysed Lys acylation in proteins

**Fig. 5:**
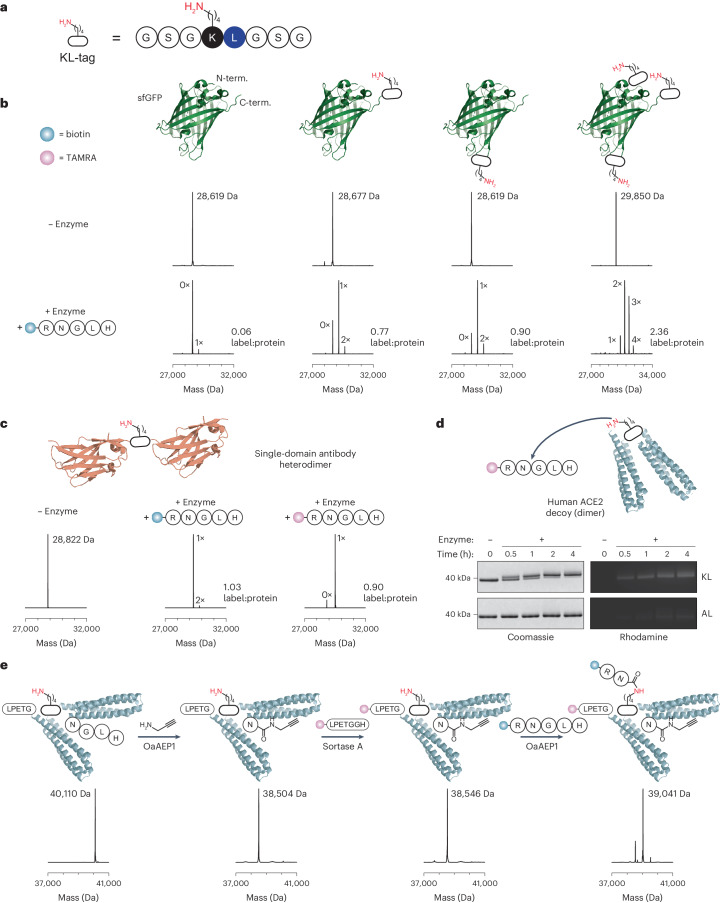
Targeted Lys acylation on protein substrates containing endogenous Lys residues. **a**, Scheme of the KL-tag, which consists of the KL recognition site flanked by 3-aa GSG linkers. **b**, Targeted Lys acylation via insertion of the KL-tag N-terminally, internally and/or C-terminally into sfGFP. Reactions were run in 100 mM HEPES buffer, pH 8, containing 5 µM sfGFP, 100 µM biotin-RNGLH, 400 µM NiSO_4_ and 0.5 µM *Oa*AEP1 for 2 h at 25 °C. Each attached biotin-RN label culminated in a mass shift of +497 Da (calc. +497 Da). Further reaction time points are shown in Extended Data Fig. 5. **c**, Labelling of a KL-tagged single-domain antibody heterodimer with biotin-RNGLH or TAMRA-RNGLH, as indicated. Reactions were run in 100 mM HEPES buffer, pH 8, containing 50 µM protein, 750 µM biotin-RNGLH or 1 mM TAMRA-RNGLH, 1 mM NiSO_4_ and 1 µM *Oa*AEP1 for 4 h at 25 °C. Mass shifts: +biotin-RN, +497 Da (calc. +497 Da); +TAMRA-RN, +682 Da (calc. +683 Da). Further reaction time points are shown in Extended Data Fig. 6 and Supplementary Fig. 21. **d**, SDS–PAGE analysis of CTC.445.2d labelling with TAMRA-RNGLH. Reactions were run in 100 mM HEPES buffer, pH 8, containing 50 µM protein (either KL-tagged or a variant where the Lys–Leu sequence was mutated to Ala–Leu), 1 mM TAMRA-RNGLH, 1 mM NiSO_4_ and 1 µM *Oa*AEP1 for the indicated time points at 25 °C. The samples run on SDS–PAGE were also analysed by ESI-MS (Extended Data Fig. 7). **e**, Triple labelling of CTC-445.2d. In the first step, *Oa*AEP1 was used to attach a C-terminal alkyne click handle via incorporation of propargylamine. Next, sortase A was used to label the protein N terminus with TAMRA-LPET. Finally, the KL-tag was acylated with biotin-RN in a second *Oa*AEP1-catalysed step. For all panels, the spectra shown are reconstructed ESI-MS spectra. The peptide label:protein ratios were calculated based on relative peak heights and are shown adjacent to the spectra. Source data

**Extended Data Fig. 5:**
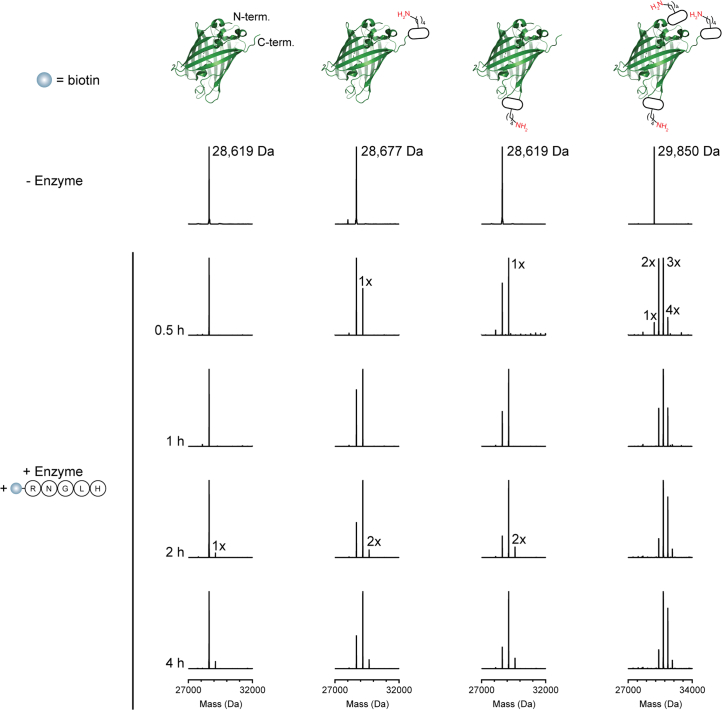
Reconstructed ESI-MS spectra for more timepoints from the sfGFP labelling experiment described in Fig. 5b. The 2 h timepoint data is also shown in Fig. 5b. Reactions were run in 100 mM HEPES buffer, pH 8, containing 5 µM sfGFP, 100 µM biotin-RNGLH, 400 µM NiSO_4_, and 0.5 µM *Oa*AEP1 for the indicated timepoints at 25 °C. Each attached biotin-RN label culminated in a mass shift of +497 Da (calc.: +497 Da).

Having demonstrated a range of peptide engineering applications, we turned our attention to the modification of recombinant proteins at internal sites. Given the minimal size of the Lys–Leu motif and thus the high likelihood of it occurring naturally in proteins, we anticipated that site-specific protein labelling would be challenging. Instead of simply inserting a Lys–Leu sequence into the protein of interest, we hypothesized that inserting a flanking linker sequence could allow for preferential labelling at that site. Given that our analysis of Lys–Leu-flanking residues revealed a Gly residue to be preferred both upstream and downstream of Lys–Leu (Extended Data Fig. 2), we selected a flexible Gly–Ser–Gly linker. We named this sequence the ‘KL-tag’ (Fig. 5a; GSG**KL**GSG) and tested its performance in superfolder GFP (sfGFP) which we deemed to be a difficult substrate due to its 20 endogenous Lys residues (Supplementary Fig. 18). Importantly, 19 of these 20 Lys residues are predicted to be solvent-exposed^42^. To suppress potential N-terminal labelling, which would interfere with our assessment of internal KL-tag labelling, we protected the protein N termini using sortase A via attachment of an N-terminally biotinylated peptide. Where the generation of an authentic N terminus is necessary, cleavage via a site-specific protease following the *Oa*AEP1-catalysed reaction could be used instead. We inserted the KL-tag either at the C terminus, within an internal loop, or simultaneously at three different sites (N terminus, C terminus and in a loop) in sfGFP (Fig. 5b). Isopeptide ligation of biotin-RNGLH to wild-type sfGFP yielded a peptide label:protein ratio of 0.06, indicating a very low level of endogenous Lys labelling (Fig. 5b and Extended Data Fig. 5). Insertion of a KL-tag C-terminally or within a surface-exposed loop enabled label:protein ratios of 0.77 or 0.90, respectively, indicating that our labelling strategy preferentially acylates Lys residues within the KL-tag. Notably, the level of site-specificity achieved for sfGFP labelling is comparable to the levels achieved with Ubc9e, an enzyme that has evolved to acylate specific Lys residues (~10–20% reported off-target labelling)^10^. We purified the single-labelled product from this mixture using size exclusion chromatography, and confirmed site-specific labelling at the KL-tag via tandem MS experiments on peptide fragments derived from tryptic, chymotryptic and endoproteinase Glu-C digests (Supplementary Figs. 19 and 20). These experiments also identified the sole off-target labelling site as the Lys residue that is located immediately upstream of the C-terminal KL-tag in a region that is probably flexible and highly accessible (Supplementary Fig. 20). We did not observe off-target labelling at the other Lys residues located in the protein core. Notably, we could also simultaneously modify sfGFP with three KL-tags in a rapid, one-pot reaction (2 h at 25 °C).

**Extended Data Fig. 6:**
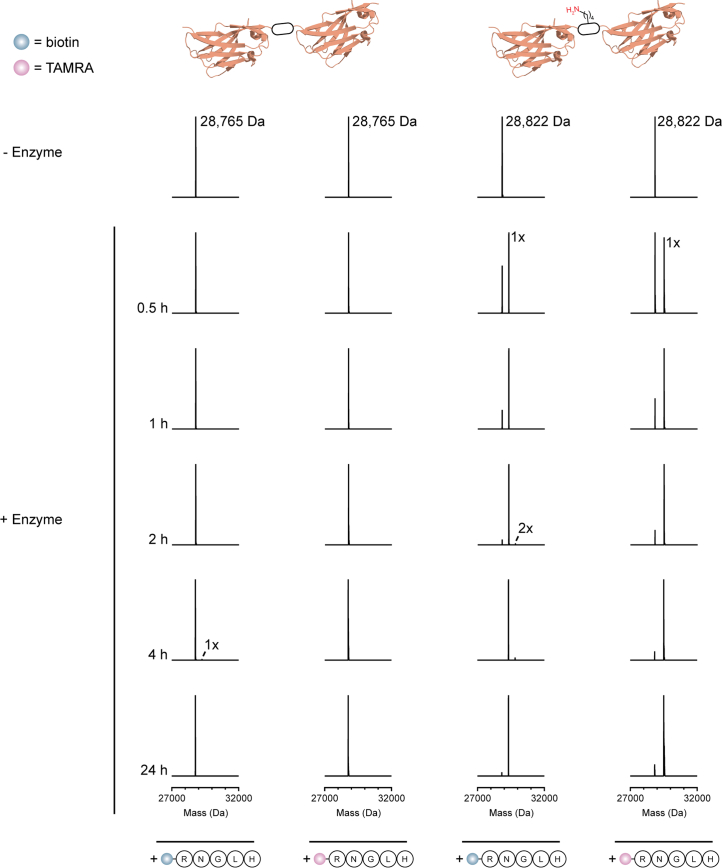
Reconstructed ESI-MS spectra for more timepoints from the single-domain antibody heterodimer labelling experiment described in Fig. 5c. The 4 h timepoint data is also shown in Fig. 5c. Timepoints for a control experiment on a protein where the Lys in the KL-tag was mutated to Ala are also shown. Reactions were run in 100 mM HEPES buffer, pH 8, containing 50 µM protein, 750 µM biotin-RNGLH or 1 mM TAMRA-RNGLH, 1 mM NiSO_4_, and 1 µM *Oa*AEP1 for the indicated timepoints at 25 °C. Mass shifts: +biotin-RN, +497 Da (calc.: +497 Da); +TAMRA-RN, +682 Da (calc.: +683 Da).

Next, we examined labelling of a single-domain antibody heterodimer (Fig. 5c) consisting of VHH_MHCII_ and VHH_6e_ (ten endogenous Lys residues). VHH_MHCII_ binds MHC class II molecules on professional antigen-presenting cells and has been used to deliver antigens for autoimmune-disorder therapies or vaccines^43,44^. VHH_6e_ binds to a 14-aa tag (6e tag) and might thus enable the plug-and-play delivery of 6e-tagged antigens^45^. The attachment of fluorescence or affinity labels to this dimer might facilitate monitoring of such experiments. We thus inserted the KL-tag within a linker that connects the two single-domain antibodies and monitored the attachment of biotin-RN or 5(6)-carboxytetramethylrhodamine (TAMRA)-RN as catalysed by *Oa*AEP1. This revealed near-quantitative conversion to the biotin– or fluorophore–protein conjugates without labelling of endogenous Lys residues in a control protein where the KL-tag Lys was mutated to an Ala (Fig. 5c, Extended Data Fig. 6 and Supplementary Fig. 21). We further confirmed labelling of the KL-tag via tandem MS analysis (Supplementary Fig. 22). We found that these reactions reached completion in 4 h, but the conjugates remained stable for at least another 20 h in the presence of *Oa*AEP1, indicating that potential reaction reversal is not problematic (Extended Data Fig. 6). Given the conserved three-dimensional (3D) folds of single-domain antibodies, we expect this strategy to be generally applicable for labelling other multimeric single-domain antibody constructs, thereby aiding the development of imaging reagents or therapeutics.

**Extended Data Fig. 7:**
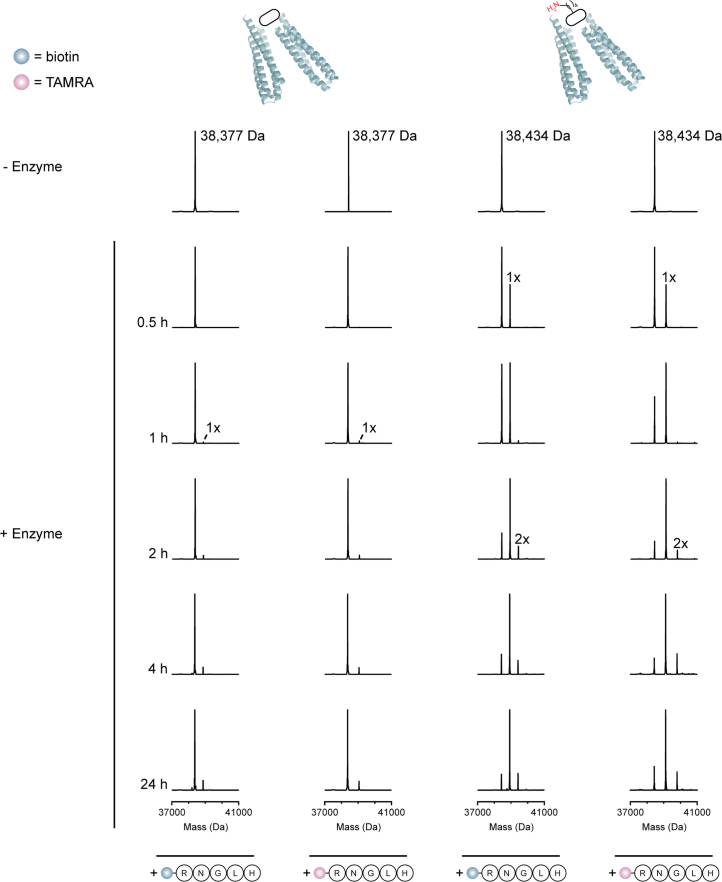
Reconstructed ESI-MS spectra for timepoints from the CTC-445.2d labelling experiment described in Fig. 5d. Shown is labelling with biotin-RNGLH or TAMRA-RNGLH. Reactions were run in 100 mM HEPES buffer, pH 8, containing 50 µM protein (either KL-tagged or a variant where the Lys-Leu sequence was mutated to Ala-Leu), 1 mM TAMRA-RNGLH or biotin-RNGLH, 1 mM NiSO_4_, and 1 µM *Oa*AEP1 for the indicated timepoints at 25 °C. Mass shifts: +biotin-RN, +497 Da (calc.: +497 Da); +TAMRA-RN, +682 Da (calc.: +683 Da).

Subsequently, we investigated labelling of a KL-tagged variant of a de novo designed, dimeric decoy protein that binds to the SARS-CoV-2 spike protein at the hACE2-interacting interface (CTC-445.2d, Fig. 5d; 30 endogenous Lys residues)^46^. We observed efficient attachment of biotin-RN or TAMRA-RN to this protein, albeit with some off-target labelling (Fig. 5d and Extended Data Fig. 7). Tandem MS analysis of the product digests revealed correct on-target labelling and two off-target labelling sites in each monomer (Supplementary Fig. 23). One of these off-target sites is an accessible Lys–Leu sequence within an α-helix and the other is a Lys in the same helix with a Leu residue in structural proximity (Supplementary Fig. 23c). Again, the product remained stable in the presence of *Oa*AEP1, even when the reactions were run for an excess 20 h (Extended Data Fig. 7).

To demonstrate the capacity of our KL-tag labelling strategy to operate with other, complementary protein labelling approaches, we investigated targeted protein triple labelling. To this end, we extended the KL-tagged dimeric hACE2 decoy N-terminally with an LPETG sortase recognition sequence and C-terminally with an *Oa*AEP1 recognition motif (NGLH) (Fig. 5e). In the first step, we used *Oa*AEP1 to irreversibly attach a C-terminal alkyne click handle via incorporation of propargylamine^21^. Next, we used sortase A to exchange the N-terminal LPETG motif with a synthetic TAMRA-LPET peptide. Finally, we attached biotin-RN to the KL-tag using *Oa*AEP1 to yield the triple-labelled protein (Fig. 5e). This demonstrates that the KL-tag labelling approach readily integrates with other protein-labelling strategies, including with other distinct *Oa*AEP1-catalysed labelling steps.

## Conclusion

In summary, we have developed an efficient enzymatic Lys acylation strategy by repurposing an enzyme that naturally performs only intramolecular transpeptidation reactions between N and C termini. Given that other enzymatic protein side chain-modifying approaches utilize enzymes that naturally catalyse highly analogous reactions (Extended Data Fig. 1), our strategy constitutes a paradigm shift in enzyme repurposing.

Our Lys acylation approach relies on the finding that internal Lys–Leu sequences can mimic canonical N-terminal Gly–Leu substrates under optimized reaction conditions. As the trace left in the product is minimal when compared to existing approaches (Extended Data Fig. 1)^10^, this strategy is highly suited to peptide engineering, which we have demonstrated both intra- and inter-molecularly via the synthesis of isopeptide-cyclized products, previously only accessible via multi-step chemical syntheses^40^, as well as dendrimer-like branching. Other enzymatic side chain-labelling strategies are largely unsuited for peptide modifications due to their bulky recognition motifs and linkages left in the product.

Furthermore, we develop KL-tags that can be preferentially acylated when inserted into recombinant proteins. KL-tags are minimal—both in terms of size and the number of bulky amino acid residues therein—when compared to other tags used for side chain labelling (Extended Data Fig. 1). The KL-tag labelling approach can readily integrate with other protein-labelling approaches, as we demonstrate for sortase A-catalysed transpeptidation, including with other distinct *Oa*AEP1-catalysed steps. The use of a single enzyme for both internal and terminal modifications in a multi-step labelling regime demonstrates the utility of *Oa*AEP1s reaction versatility.

Overall, our approach is compatible with proteinogenic substrates, proceeds under mild, aqueous conditions, and only requires sub-stoichiometric quantities of enzyme. Our Article demonstrates enzymatically linking a peptide with an authentic C terminus to a targeted Lys side chain within a minimal recognition motif in a single step. Thus, *Oa*AEP1-catalysed Lys acylation is a valuable addition to the protein and peptide engineer’s toolbox.

## Methods

### Reagents

All reagents were obtained from commercial sources and used as purchased: dichloromethane (DCM), *N*,*N*-diisopropylethylamine (DIPEA) and methanol (MeOH) from MiliporeSigma; dimethylformamide (DMF), diethyl ether and acetonitrile from RCI Labscan; piperidine, acetic anhydride and trifluoroacetic acid (TFA) from Chem-Supply; HATU from ChemImpex Int; HCTU from CSBio; ethylenediamine (Eda), triisopropylsilane (TIS), iodine flakes, glacial acetic acid and biotin from Sigma; 5(6)-carboxytetramethylrhodamine (5(6)-TAMRA) from ChemPep. All Fmoc-protected amino acids were supplied from CSBio, except for Fmoc-d-Phe-OH from Merck. Water was purified using a Milli-Q direct water-purification system.

### Peptide synthesis

Following standard Fmoc-solid-phase peptide synthesis (Fmoc-SPPS) protocols, all peptide sequences were synthesized on 2-chlorotrityl chloride resin (0.45 mmol g^−1^, CSBio) at a 125 µmol scale, using an automated Symphony multiplex peptide synthesizer (Protein Technologies). Unless noted otherwise, all reactions were carried out at room temperature and purified by preparative and/or semipreparative RP-HPLC. Preparative RP-HPLC was performed using a Phenomenex Gemini C18 column (5 µm, 110 Å, 250 × 21.2 mm) at a flow rate of 8 ml min^−1^. Semipreparative RP-HPLC was performed using a Phenomenex Gemini C18 column (5 µm, 110 Å, 250 × 10 mm) at a flow rate of 3 ml min^−1^. Analytical RP-HPLC was performed using a Jupiter C18 column (5 µm 300 Å, 250 × 4.6 mm) at a flow rate of 1 ml min^−1^.

#### General resin loading and capping protocol

The resin was first swollen in dry DCM for 1 h and then washed with DMF. A solution of protected amino acid (4 equiv.) and DIPEA (8 equiv.) in 2 ml of DMF was then added to the resin and agitated. After 12 h, the resin was washed with DMF, DCM and DMF. Unreacted resin sites were then capped by adding a freshly prepared solution of DCM/MeOH/DIPEA (17:2:1, vol/vol/vol, 5 ml) to the resin and agitating for 30 min. Following capping, the resin was washed with DMF, DCM and DMF. Peptide assembly was then performed in accordance with the standard Fmoc-SPPS protocols described in the following.

#### Synthesis of C-terminal Eda peptides

The resin was first swollen in dry DCM for 1 h and then washed with DMF. The resin was then treated with a freshly prepared solution of 10% Eda (vol/vol) and DIPEA (2 equiv. relative to resin loading) in DMF and agitated overnight. After 12 h, the resin was washed with DMF, DCM and DMF. Peptide assembly was then performed in accordance with the standard Fmoc-SPPS protocols given in the following.

#### Standard peptide assembly (Fmoc-SPPS)

The following general automated protocol of peptide elongation was followed for canonical and non-canonical amino acids using iterative Fmoc-SPPS, unless stated otherwise:

1. **Deprotection.**The resin was treated with 30% piperidine in DMF (3 ml, 2 × 3 min) and then washed with DMF.
2. **General amino acid coupling.**A solution of protected amino acid (2 equiv.), HCTU (2 equiv.) and DIPEA (4 equiv.) in 4 ml of DMF was added to the resin and the reaction allowed to proceed for 10 min. The coupling was repeated by draining the resin and adding fresh coupling solution. After 10 min the resin was washed with DMF.

#### N-terminal amine acetylation

Following the final amino acid coupling and subsequent Fmoc deprotection, the resin was treated with a solution of DMF, DIPEA and acetic anhydride (17:2:1 vol/vol/vol, 5 ml; 2 × 3 min) and then washed with DMF.

#### N-terminal biotinylation

Following the final amino acid coupling and subsequent Fmoc deprotection, a pre-activated solution of biotin (3 equiv. relative to resin amine loading), HATU (2.5 equiv.) and DIPEA (6 equiv.) in 4 ml of DMF was added to the resin and agitated. After 2 h, the resin was washed with DMF, DCM and DMF, and the crude peptide was cleaved and purified in accordance with the protocols described below.

#### N-terminal 5(6)-TAMRA conjugation to peptides

Following the final amino acid coupling and subsequent Fmoc deprotection, a pre-activated solution of 5(6)TAMRA (3 equiv. relative to resin amine loading), HATU (2.5 equiv.) and DIPEA (6 equiv.) in 2 ml of DMF was added to the resin and agitated under protection from ambient light. After 2 h, the resin was washed with DMF, DCM and DMF, and the crude peptide was cleaved and purified in accordance with the protocols described above, while mitigating exposure to ambient light. During the purification process, the two TAMRA isomers were separated and only one was used for the labelling reactions.

#### Cleavage

A mixture of TFA/TIS/water (95:2.5:2.5 vol/vol/vol, 5 ml) was added to the resin. After 2 h, the resin was drained and washed with TFA (5 ml).

#### Work-up

The combined TFA cleavage solutions were concentrated under a stream of N_2_. The remaining residue was treated with pre-chilled diethyl ether to precipitate the crude peptide, which was subsequently dissolved in water and acetonitrile containing 0.1% TFA, filtered and purified by RP-HPLC with a gradient of 0–50% in 45 min. Fractions containing the target product mass at the highest purity (>95%) were collected and lyophilized. Peptide purity was assessed by analytical RP-HPLC (Phenomenex Jupiter 5 μm C18 300 Å, 20 min 0–60% acetonitrile gradient).

#### Cysteine oxidation

Following an initial RP-HPLC purification step, the fractions containing the reduced peptide were diluted tenfold in Milli-Q water and treated with a concentrated solution of iodine in glacial acetic acid, added dropwise until a persistent pale-yellow colour was reached. The reaction was left at room temperature for 10 min. The reaction was then quenched with ascorbic acid and purified via RP-HPLC with a gradient of 0–50% in 45 min. Fractions containing target product mass at the highest purity (>95%) were collected and lyophilized. Peptide purity was assessed via analytical RP-HPLC (Phenomenex Jupiter 5 μm C18 300 Å, 20 min 0–60% acetonitrile gradient).

### Reaction temperature

All reactions described as run at 25 °C were run at room temperature.

### CD spectroscopy

CD spectroscopy was used to estimate the overall secondary structure content of the truncated stinging tree-derived crambin-like peptides. Peptide samples were prepared in aqueous CD buffer (100 mM NaF, 10 mM KH_2_PO_4_, pH 7.5) at a concentration of 60 µM. CD spectra were collected and averaged from five scans, with 1-mm-path-length cuvettes at wavelengths from 190 to 260 nm with 1-nm data intervals, using a Jasco J-810 spectropolarimeter. Blanks were subtracted, and the mean residue ellipticity (*θ*) was calculated from the CD output response unit in degrees. Percentage peptide helicity was determined using the Luo–Baldwin formula using the value between 218 and 222 nm and converted as previously described^47^.

### NMR spectroscopy

NMR spectroscopy was performed on a Bruker Avance III 600-MHz spectrometer equipped with a cryogenically cooled probe. Peptides purified by RP-HPLC were dissolved in 500 μl of H_2_O/D_2_O (9:1) at a peptide concentration of ~1 mM. NMR experiments including 2D TOCSY (80-ms mixing time) and NOESY (200-ms mixing time) were acquired at 298 K and used to sequentially assign backbone and side chain protons. Solvent suppression was achieved using excitation sculpting and spectra were referenced to water at 4.77 ppm. All spectra were processed using Topspin v3.6 and assigned using CCPNMR Analysis.

### RP-HPLC-based assessment of reactions

Reactions, conducted as detailed in the figure legends (where GLH was quenched for orientation control, reactions were incubated at 25 °C for 15 min before enzyme addition; Fig. 3c), were quenched by TFA addition to 1% vol/vol and resolved on an analytical Phenomenex Jupiter 5 µm C18 300-Å column. Relative peak integrals were used to calculate the extent of conversion to product. Peptide concentrations were determined by weight under the assumption that charged residues and free N termini carried TFA salt.

### MS

Reactions of peptide substrates were analysed by MALDI-TOF MS (SCIEX 5800 MALDI–TOF-MS, α-cyano-4-hydroxycinnamic acid matrix). Protein-labelling reactions were loaded on a Zorbax 300SB-C18 column (Agilent) and eluted using a Shimadzu Nexera X2 LC system over a 15-min 1–50% acetonitrile gradient. The liquid chromatography outflow was connected to a QSTAR Elite system (SCIEX). Reconstructed spectra were generated in Analyst software (SCIEX).

To identify labelling sites, digests of protein-labelling reactions were analysed by LC-MS/MS, where 50-µl completed labelling reactions (labelled with biotin-RN; set in at least triplicate) were diluted to 500 µl with NH_4_HCO_3_ (100 mM, pH 8) and concentrated in an Amicon spin column to remove excess biotin-RNGLH peptide. Each reaction replicate was then split into three tubes for separate digestion with trypsin (Promega), chymotrypsin (Sigma) and endoproteinase Glu-C (Sigma) (1 µg for each enzyme) at 37 °C for 18 h. Samples were quenched by adding formic acid to a final concentration of 3% (vol/vol), then loaded on a Zorbax 300SB-C18 column (Agilent) and eluted using a gradient of 1–70% acetonitrile over 71 min on a Shimadzu Nexera X2 LC system. The outflow was interfaced with a 5600 Triple TOF mass spectrometer (SCIEX). The resultant MS/MS data files were then combined and analysed via ProteinPilot using the ‘none’ enzyme digestion option, a database containing the expected protein sequence, and custom modification settings for biotin–Arg–Asn- or Asn-modified lysines.

### Molecular modelling

The 3D structures of *Oa*AEP1 (S-acyl intermediate) in complex with a peptide substrate, either GLGV-NH_2_ or Ac-GKLGV-NH_2_ and denoted OaAEP-G and OaAEP-K, respectively, were modelled by homology using Modeller 10v4 (ref. ^48^). The structures used as templates were a crystal structure of a C214A mutant of VyPAL2 bound to the N-terminal polypeptide tail from a neighbouring ligase in the crystal lattice^49^ (PDB 7F5P) and a model of a thioester intermediate (INT1) of a legumain that occurs along the proteolysis pathway^50^. For OaAEP-K, the distance between NZ(substrate Lys) and C(thioester Asn) was restrained using a harmonic potential during model generation. The succinimide residue of OaAEP was modelled as a rigid body (BLK). A hundred models were generated, and the model with the lowest DOPE score was selected for further computation using NAMD 2.14 (ref. ^51^). CHARMM36 was parameterized to include a succinimide and a thioester link. All His residues were modelled in a neutral state with the proton on NE2. The systems were solvated in a water box and ions added to reach neutrality. The systems were then relaxed using 5,000-step conjugate gradient minimization and equilibrated over 100 ps of constant temperature (298 K) and then 100 ps with pressure regulation at 1 atm using a Langevin piston, while restraining all non-hydrogen atoms constraining the distance between the N (substrate Gly for OaAEP-G) or NZ (substrate Lys, OaAEP-K) and C (thioester Asn). The restraints were then progressively released by constraining only the backbone atoms and the active site, then secondary structure elements and the active site, while keeping the distance restraint between the substrate and the thioester over 200 ps. The systems were further relaxed by gradually releasing the distance restraint between the substrate and the thioester over 4 ns, and then studied over 25 ns of unrestrained molecular dynamics simulations. Molecular dynamics simulations from system equilibration to unrestrained simulations were performed in triplicate.

### Protein production and purification

Protein substrates and *Oa*AEP1 were produced in *Escherichia coli* BL21 or SHuffle, as previously reported by us^23^. *Oa*AEP1 and the single-domain antibody heterodimer were produced in SHuffle; all other proteins were produced in BL21. Plasmids (pHUE for the enzyme, pET14b for all other proteins) were transformed into the respective strains and expression was induced with 0.3 mM isopropyl β-d-1-thiogalactopyranoside (IPTG) after growth at 37 °C (BL21) or 30 °C (SHuffle), with shaking at 200 r.p.m. in Luria Bertani broth (100 µg ml^−1^ ampicillin), until reaching an optical density at 600 nm of 0.6–0.8. Following induction, the cells were grown overnight at 18 °C, then harvested via centrifugation, suspended in lysis buffer (50 mM NaH_2_PO_4_, 300 mM NaCl, 10 mM imidazole, pH 8) and lysed at 32 kpsi using a Constant Systems cell disruptor. Cell lysates were clarified via centrifugation at 40,000*g* and soluble protein was bound to NiNTA beads (Qiagen), washed (50 mM NaH_2_PO_4_, 300 mM NaCl, 20 mM imidazole, pH 8) and eluted (50 mM NaH_2_PO_4_, 300 mM NaCl, 250 mM imidazole, pH 8). Substrate proteins were then cleaved at TEV (ENLYFQ), Factor Xa (IEGR) and/or sortase A (LPETG) sites by addition of the corresponding enzyme(s). We used the heptamutant version of sortase A (SrtA-7M)^52^ and accelerated cleavage by adding 10 mM benzylamine in 100 mM HEPES buffer, pH 8. To simplify the analysis of our reactions, we labelled protein substrates N-terminally using SrtA-7M (~50 µM protein, 0.8–2 mM biotin-LPETGGH, 250 µM NiSO_4_, 1 µM SrtA-7M, 100 mM HEPES buffer, pH 8) following an NAP-5 buffer exchange step. Subsequently, flowthrough from rebinding the substrates to Ni-NTA was collected. Where necessary, proteins were further purified by size exclusion chromatography (SEC) on a HiLoad 16/600 Superdex 75-pg column equilibrated with HEPES buffer (pH 8) (substrates) or 20 mM Tris buffer containing 100 mM NaCl and 10% glycerol (pH 8) (enzyme). When substrate proteins were not additionally purified by SEC, they were buffer-exchanged by NAP-5 into 100 mM HEPES buffer, pH 8. Auto-activation of the enzyme was carried out via addition of acetic acid (~1:500 vol/vol acid:buffer, to pH 4.0–4.5) and incubation at 37 °C for 2–4 h. Protein substrate and enzyme concentrations were determined via NanoDrop A_280_ readings using calculated protein extinction coefficients and molecular weights. All proteins were frozen in liquid N_2_ and stored at −80 °C until use.

### Protein triple labelling

Protein triple labelling was demonstrated with CTC-445.2d modified with an N-terminal LPETG, an internal KL-tag and a C-terminal NGLH motif. In the first step, 50 µM protein was C-terminally labelled using 1 µM *Oa*AEP1 in 100 mM HEPES buffer, pH 7, containing 20 mM propargylamine. Next, the reaction was diluted tenfold with 100 mM HEPES buffer, pH 8, and bound to Ni-NTA. After collecting the flowthrough, the protein was N-terminally cleaved with SrtA-7M in the presence of 5 mM propargylamine. Next the protein was buffer-exchanged into 100 mM HEPES buffer, pH 8, using NAP-5, and N-terminally labelled in a reaction containing 30 µM protein, 450 µM TAMRA-LPETGGH and 1 µM SrtA-7M. The SrtA-7M was subsequently removed by rebinding to Ni-NTA and collecting the flowthrough. Next, the reaction was buffer-exchanged via NAP-5 into 100 mM HEPES buffer, pH 8, and the KL-tag labelling was carried out in a reaction containing 50 µM protein, 1 mM biotin-RNGLH, 1 mM NiSO_4_ and 1 µM *Oa*AEP1 for 4 h at 25 °C.

### SDS–PAGE

For analysis by SDS–PAGE, proteins were boiled in reducing loading dye before being run on NuPAGE 4–12% Bis-Tris gels. The gels were imaged for rhodamine fluorescence using a BioRad Gel Doc, stained with InstantBlue, and then imaged again for Coomassie staining. Identical fluorescence exposure times and analyses were performed for the different gels. Images were analysed using the BioRad Image Lab software. The contrast for the rhodamine fluorescence images in Fig. 5d were adjusted by the editorial team and Supplementary Fig. 21 was adjusted in the same way.

## Online content

Any methods, additional references, Nature Portfolio reporting summaries, source data, extended data, supplementary information, acknowledgements, peer review information; details of author contributions and competing interests; and statements of data and code availability are available at 10.1038/s41557-024-01520-1.

## Supplementary information

Supplementary Information

Supplementary Data 1 DNA sequences of constructs expressed in this study.

Supplementary Data 2 Molecular dynamics initial and final coordinates.

## Source data

Source Data Fig. 3 Statistical source data.

Source Data Fig. 5 Unprocessed gels.

Source Data Extended Data Fig./Table 2 Statistical source data.

Source Data Extended Data Fig./Table 3 Statistical source data.

Source Data Extended Data Fig./Table 4 Statistical source data.

## Data Availability

All data supporting the findings of this study are included in the Article, Supplementary Information and source data files. Images of uncropped gels are provided as Source Data and in the Supplementary Information. Source data are provided with this paper.
