# Author Correction: Repurposing a plant peptide cyclase for targeted lysine acylation

**DOI:** 10.1038/s41557-024-01625-7

**Published:** 2024-08-13

**Authors:** Fabian B. H. Rehm, Tristan J. Tyler, Yan Zhou, Yen-Hua Huang, Conan K. Wang, Nicole Lawrence, David J. Craik, Thomas Durek

**Affiliations:** Institute for Molecular Bioscience, Australian Research Council Centre of Excellence for Innovations in Peptide and Protein Science, The University of Queensland, Brisbane, Queensland Australia

**Keywords:** Ligases, Peptides

Correction to: *Nature Chemistry* 10.1038/s41557-024-01520-1, published online 24 May 2024.

In the version of the article initially published, in Fig. 3f the *x* axis read “Retention time (min)”, which has now been corrected to “*m*/*z*”, while the x-axis units have been updated from “14, 15, 16” to “2,500, 3,000, 3,500” in the HTML and PDF versions of the article.

