# Supplementary material for: Repurposing a plant peptide cyclase for targeted lysine acylation
Source: Nat Chem. 2024 May 24;16(9):1481–9. doi: 10.1038/s41557-024-01520-1 (PMC11374674; doi:10.1038/s41557-024-01520-1)
Supplement: Supplementary file 1 — Supplementary Information [file 41557_2024_1520_MOESM1_ESM.pdf]

nature chemistry

Article

https://doi.org/10.1038/s41557-024-01520-1

Repurposing a plant peptide cyclase for targeted lysine acylation

In the format provided by the authors and unedited

Contents:

1. **Supplementary tables** **page S2**
2. **RP-HPLC chromatograms of synthetic peptides** **page S10**
3. **Full sequences of protein substrates** **page S13**
4. **Supplementary figures** **page S14**
5. **Unprocessed gel images** **page S37**
6. **Supplementary references** **page S38**

1. Supplementary tables

Supplementary table 1. Calculated and observed masses of peptides used in the main figures. Observed masses were measured via ESI LC-MS.

Peptide sequences	Calculated $[M+H]^+$ (Da)	Observed $[M+H]^+$ (Da)
Ac-RWRGWRNGLH*	1379.7	1380.0
Biotin-RNGLH*	822.4	822.6
TAMRA-RNGLH	1008.5	1008.3
Ac-GAKGV	473.3	473.3
Ac-GLKGV	515.3	515.3
Ac-GKAGV	473.3	473.3
Ac-GKLGV	515.3	515.3
Ac-GLKLGV	628.4	628.4
Ac-GCGSKLGSCGHfRWGSNGLH (disulfide bond between the two C)	2099.9	2100.4
Ac-GCGSKLGSCGHfRWGSNGLH	2101.9	2102.4
Ac-GCGSALGSCGHfRWGSNGLH (disulfide bond between the two C)	2042.9	2043.5
GLHGCGSKLGSCGHfRWGSNGLH (disulfide bond between the two C)	2365.1	2365.6
Ac-GSTTLKNIYNTCRFGGGSRTLCARLSGNGLH (disulfide bond between the two C)	3294.6	3295.4
Ac-GSTTAKNIYNTCRFGGGSRTLCARLSGNGLH (disulfide bond between the two C)	3252.6	3253.3
GLHGKLGRL- Eda	992.6	992.5
Biotin-LPETGGH	936.4	936.4
TAMRA-LPETGGH	1122.5	1122.5

] = disulfide bond

*Also used by us in previous work[1-3]

Supplementary table 2. Calculated and observed masses for the **KX** peptides. Observed masses were measured via ESI LC-MS.

Peptide sequences	Calculated $[M+H]^+$ (Da)	Observed $[M+H]^+$ (Da)
Ac-AG**KL**GA	558.3	558.4
Ac-AG**KI**GA	558.3	558.3
Ac-AG**KV**GA	544.3	544.3
Ac-AG**KF**GA	592.3	592.3
Ac-AG**KM**GA	576.3	576.3
Ac-AG**KR**GA	601.3	601.3
Ac-AG**KE**GA	574.3	574.3
Ac-AG**KG**GA	502.3	502.3
Ac-AG**KP**GA	542.3	542.3
Ac-AG**KH**GA	582.3	582.3
Ac-AG**KQ**GA	573.3	573.3
Ac-AG**KY**GA	608.3	608.3
Ac-AG**KW**GA	631.3	631.3
Ac-AG**KA**GA	516.3	516.3
Ac-AG**KS**GA	532.3	532.3

Supplementary table 3. Calculated and observed masses for the **XKL** peptides. Observed masses were measured via ESI LC-MS.

**Peptide sequences**	**Calculated $[M+H]^+$ (Da)**	**Observed $[M+H]^+$ (Da)**
Ac-A**LKL**GA	614.4	614.4
Ac-A**IKL**GA	614.4	614.4
Ac-A**VKL**GA	600.4	600.4
Ac-A**FKL**GA	648.4	648.4
Ac-A**MKL**GA	632.3	632.4
Ac-A**RKL**GA	657.4	657.4
Ac-A**EKL**GA	630.3	630.4
Ac-A**GKL**GA	558.3	558.4
Ac-A**PKL**GA	598.3	598.4
Ac-A**HKL**GA	638.4	638.4
Ac-A**QKL**GA	629.4	629.4
Ac-A**YKL**GA	664.4	664.4
Ac-A**WKL**GA	687.4	687.4
Ac-A**AKL**GA	572.3	572.4
Ac-A**SKL**GA	588.3	588.4

Supplementary table 4. Calculated and observed masses for the **KLX** peptides. Observed masses were measured via ESI LC-MS.

**Peptide sequences**	**Calculated $[M+H]^+$ (Da)**	**Observed $[M+H]^+$ (Da)**
Ac-AG**KLL**A	614.4	614.4
Ac-AG**KLI**A	614.4	614.4
Ac-AG**KLV**A	600.4	600.4
Ac-AG**KLF**A	648.4	648.4
Ac-AG**KLM**A	632.3	632.3
Ac-AG**KLR**A	657.4	657.4
Ac-AG**KLE**A	630.3	630.4
Ac-AG**KLG**A	558.3	558.4
Ac-AG**KLP**A	598.3	598.4
Ac-AG**KLH**A	638.4	638.4
Ac-AG**KLQ**A	629.4	629.4
Ac-AG**KLY**A	664.4	664.4
Ac-AG**KLW**A	687.4	687.4
Ac-AG**KLA**A	572.3	572.4
Ac-AG**KLS**A	588.3	588.4

Supplementary table 5. Calculated and observed masses for the model NGLH-containing peptide substrate (Ac-RWRGWRNGLH) and the conjugate Ac-AG**KX**GA products. Observed masses were measured via ESI LC-MS.

Peptide conjugate sequences	Calculated $[M+H]^+$ (Da)	Observed $[M+H]^+$ (Da)
Unmodified substrate	1379.7	1380.0
Hydrolysis	1072.5	1072.6
Ac-AG**KL**GA	1611.9	1612.1
Ac-AG**KI**GA	1611.9	1612.1
Ac-AG**KV**GA	1597.8	1598.1
Ac-AG**KF**GA	1645.8	1646.1
Ac-AG**KM**GA	1629.8	1630.0
Ac-AG**KR**GA	1654.9	1655.1
Ac-AG**KE**GA	1627.8	1628.0
Ac-AG**KG**GA	1555.8	1556.0
Ac-AG**KP**GA	1595.8	1596.0
Ac-AG**KH**GA	1635.8	1636.0
Ac-AG**KQ**GA	1626.8	1627.0
Ac-AG**KY**GA	1661.8	1662.0
Ac-AG**KW**GA	1684.8	1685.1
Ac-AG**KA**GA	1569.8	1570.0
Ac-AG**KS**GA	1585.8	1586.0

Supplementary table 6. Calculated and observed masses for the model NGLH-containing peptide substrate (Ac-RWRGWRNGLH) and the conjugate Ac-A**XKL**GA products. Observed masses were measured via ESI LC-MS.

Peptide conjugate sequences	Calculated $[M+H]^+$ (Da)	Observed $[M+H]^+$ (Da)
Unmodified substrate	1379.7	1380.0
Hydrolysis	1072.5	1072.6
Ac-A**LKL**GA	1667.9	1668.1
Ac-A**IKL**GA	1667.9	1668.2
Ac-A**VKL**GA	1653.9	1654.2
Ac-A**FKL**GA	1701.9	1702.2
Ac-A**MKL**GA	1685.9	1686.2
Ac-A**RKL**GA	1710.9	1711.2
Ac-A**EKL**GA	1683.9	1684.2
Ac-A**GKL**GA	1611.9	1612.2
Ac-A**PKL**GA	1651.9	1652.1
Ac-A**HKL**GA	1691.9	1692.1
Ac-A**QKL**GA	1682.9	1683.1
Ac-A**YKL**GA	1717.9	1718.1
Ac-A**WKL**GA	1740.9	1741.1
Ac-A**AKL**GA	1625.9	1626.1
Ac-A**SKL**GA	1641.9	1642.1

Supplementary table 7. Calculated and observed masses for the model NGLH-containing peptide substrate (Ac-RWRGWRNGLH) and the conjugate Ac-AG**KLX**A products. Observed masses were measured via ESI LC-MS.

**Peptide conjugate sequences**	**Calculated $[M+H]^+$ (Da)**	**Observed $[M+H]^+$ (Da)**
Ac-AG**KLL**A	1379.7	1380.0
Ac-AG**KLI**A	1072.5	1072.6
Ac-AG**KLV**A	1667.9	1668.2
Ac-AG**KLF**A	1667.9	1668.3
Ac-AG**KLM**A	1653.9	1654.3
Ac-AG**KLR**A	1701.9	1702.2
Ac-AG**KLE**A	1685.9	1686.2
Ac-AG**KLG**A	1710.9	1711.3
Ac-AG**KLP**A	1683.9	1684.2
Ac-AG**KLH**A	1611.9	1612.1
Ac-AG**KLQ**A	1651.9	1652.1
Ac-AG**KLY**A	1691.9	1692.1
Ac-AG**KLW**A	1682.9	1683.1
Ac-AG**KLA**A	1717.9	1718.1
Ac-AG**KLS**A	1740.9	1741.2
Ac-AG**KLL**A	1625.9	1626.1
Ac-AG**KLI**A	1641.9	1642.1

Supplementary table 8. Calculated and observed masses for products from the NGLH-containing peptide substrate reactions. Observed masses were measured via MALDI-TOF MS.

**Peptide conjugate sequences**		**Calculated [M+H]$^+$ (Da)**	**Observed [M+H]$^+$ (Da)**
Ac-GCGSKLGSCGHfRWGSNGLH			
	Hydrolysis	1792.8	1793.2
	Isopeptide-cyclized	1774.7	1775.2
Ac-GCGSKLGSCGHfRWGSNGLH			
	Hydrolysis	1794.8	1795.2
	Isopeptide-cyclized	1776.8	1777.2
Ac-GCGSALGSCGHfRWGSNGLH			
	Hydrolysis	1735.7	1736.2
	Isopeptide-cyclized	n/a	n/a
GLHGCGSKLGSCGHfRWGSNGLH			
	Hydrolysis	2057.9	2058.4
	Head-to-tail cyclized	2039.9	2040.3
	Isopeptide-cyclized	2039.9	2040.3
Ac-GSTTLKNIYNTCRFGGGSRTLCARLSGNGLH			
	Hydrolysis	2987.5	2988.0
	Isopeptide-cyclized	2969.4	2971.0
Ac-GSTTAKNIYNTCRFGGGSRTLCARLSGNGLH			
	Hydrolysis	2945.4	2946.0
	Isopeptide-cyclized	2927.4	2928.0

2. RP-HPLC chromatograms of synthetic peptides

Ac-RWRGWRNGLH

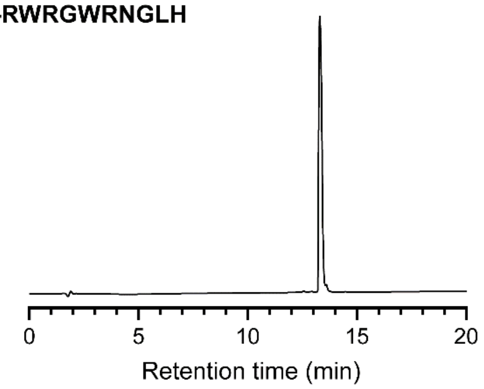

Ac-GLKGV

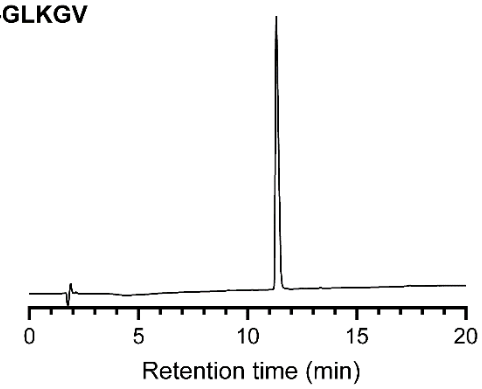

biotin-RNGLH

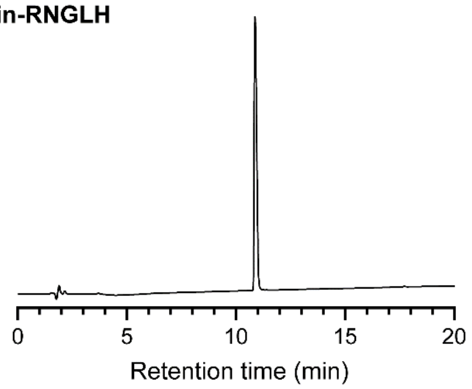

Ac-GKAGV

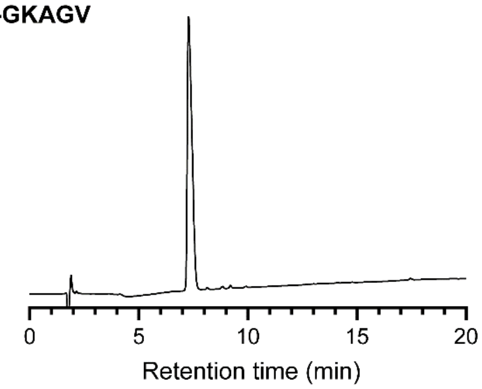

5(6)TAMRA-RNGLH

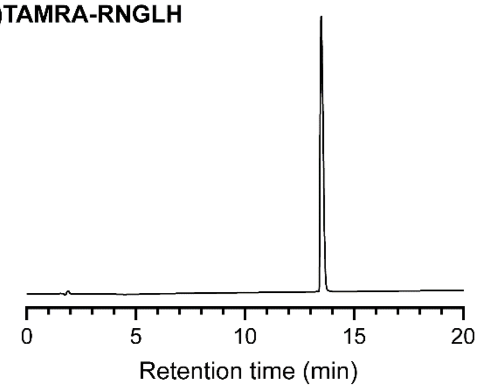

Ac-GKLGV

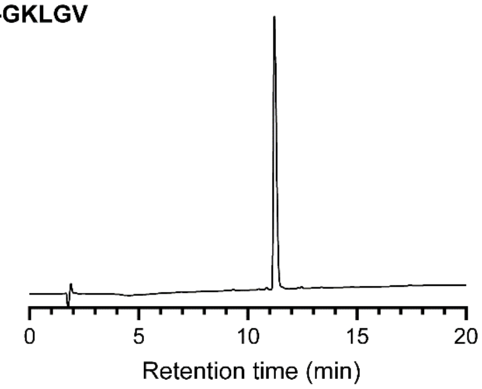

Ac-GAKGV

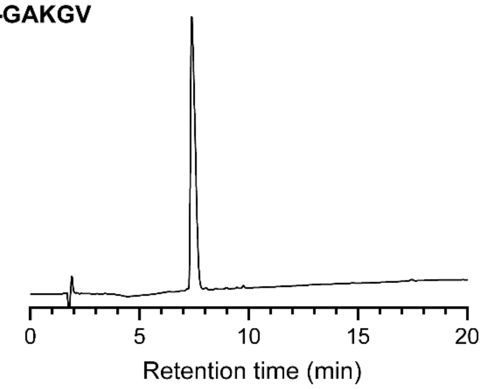

Ac-GLKLGV

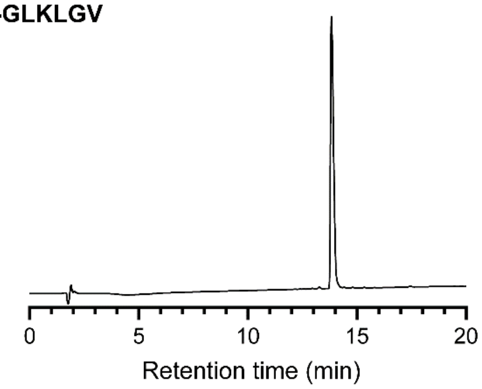

Ac-GCGSKLGSCGHfRWGSNGLH

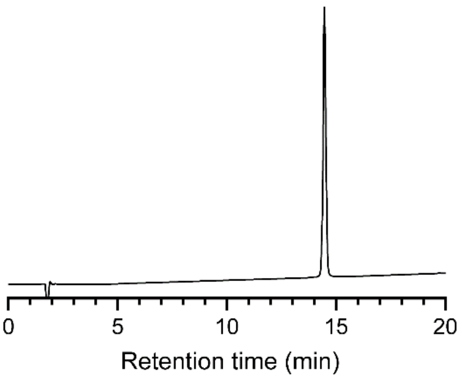

Ac-GSTTLKNIYNTCRFGGGSRTLCARLSGNGLH

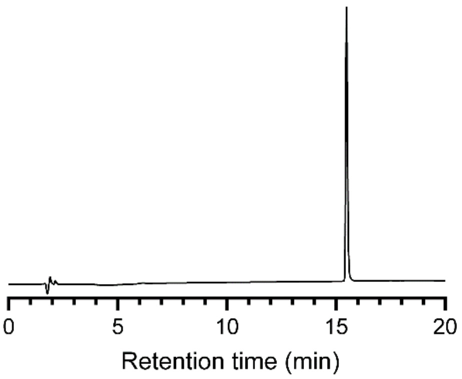

Ac-GCGSKLGSCGHfRWGSNGLH

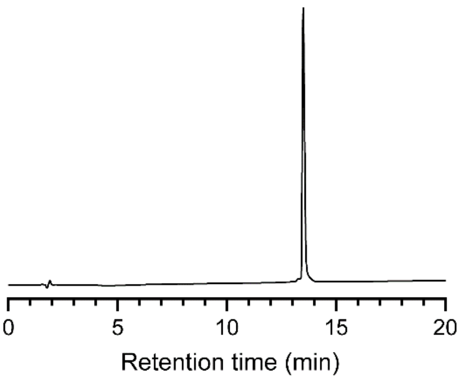

Ac-GSTTAKNIYNTCRFGGGSRTLCARLSGNGLH

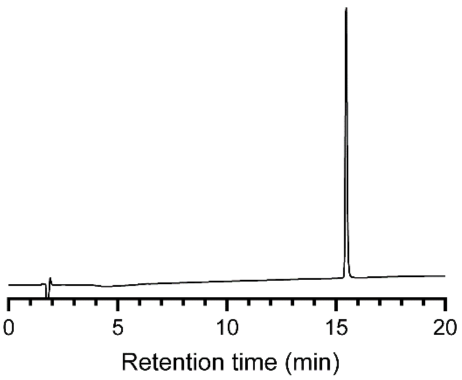

Ac-GCGSALGSCGHfRWGSNGLH

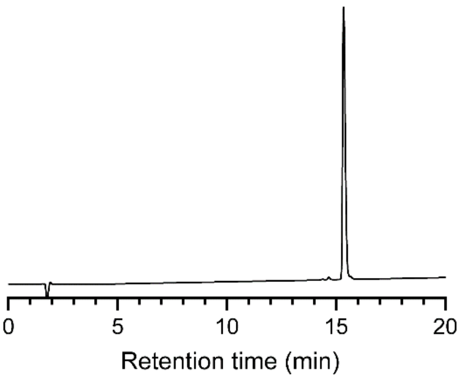

GLHGKLGRL-Eda

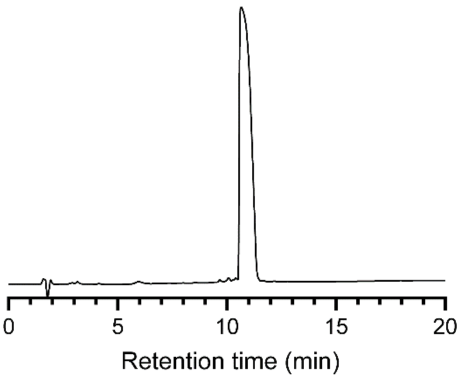

GLHGCGSKLGSCGHfRWGSNGLH

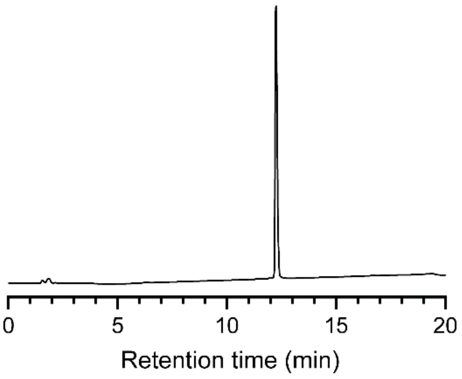

biotin-LPETGGH

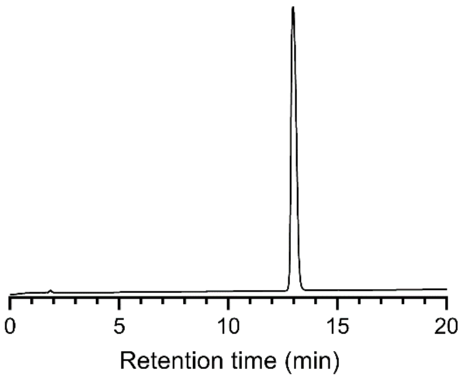

5(6)TAMRA-LPETGGH

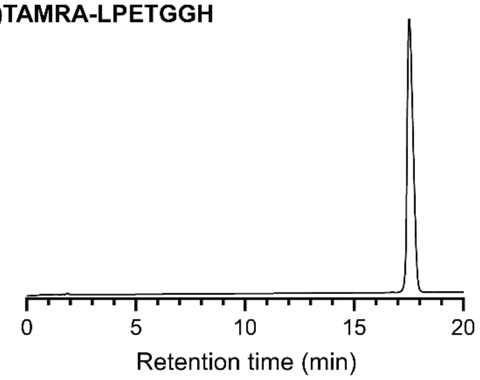

3. Full sequences of protein substrates

(calculated and observed masses (Da) for the underlined sequences below with an N-terminal biotin-LPET sequence unless otherwise stated):

sfGFP negative control (calc.: 28,620; obs.: 28,619)
MPLPETGGVSRKGEELFTGVVPILVELDGDVNGHKFSVRGEGEGDATNGKLTLKFICTTGKLPVPWPTLVTTLTYGVQCFARY
PDHMKQHDFFKSAMPEGYVQERTISFKDDGTYKTRAEVKFEGDTLVNRIELKGIDFKEDGNILGHKLEYNFNSHNVYITADKQ
KNGIKANFKIRHNVEDGSVQLADHYQQNTPIGDGPVLLPDNHYLSTQSVLSKDPNEKRDHMVLLEFVTAAGITHGMAELYKGS
GALGSGIEGRGHHHHHH

sfGFP C-terminal KL-tag (calc.: 28,677; obs.: 28,677)
MPLPETGGVSRKGEELFTGVVPILVELDGDVNGHKFSVRGEGEGDATNGKLTLKFICTTGKLPVPWPTLVTTLTYGVQCFARY
PDHMKQHDFFKSAMPEGYVQERTISFKDDGTYKTRAEVKFEGDTLVNRIELKGIDFKEDGNILGHKLEYNFNSHNVYITADKQ
KNGIKANFKIRHNVEDGSVQLADHYQQNTPIGDGPVLLPDNHYLSTQSVLSKDPNEKRDHMVLLEFVTAAGITHGMAELYK**GS**
GKLGSGIEGRGHHHHHH

sfGFP internal KL-tag (calc.: 28,620; obs.: 28,619)
MPLPETGGVSRKGEELFTGVVPILVELDGDVNGHKFSVRGEGEGDATNGKLTLKFICTTGKLPVPWPTLVTTLTYGVQCFARY
PDHMKQHDFFKSAMPEGYVQERTISFKDDGTYKTRAEVKFEGDTLVNRIELKGIDFKEDGNILGHKLEYNFNSHNVYITADKQ
KNGIKANFKIRHNVED**GSGKLGSG**SVQLADHYQQNTPIGDGPVLLPDNHYLSTQSVLSKDPNEKRDHMVLLEFVTAAGITHGM
AELYKIEGRGHHHHHH

sfGFP with N-terminal, internal and C-terminal KL-tags (calc.: 28,851; obs.: 28,850)
MPVENLYFQG**GSGKLGSG**VSRKGEELFTGVVPILVELDGDVNGHKFSVRGEGEGDATNGKLTLKFICTTGKLPVPWPTLVTTL
TYGVQCFARYPDHMKQHDFFKSAMPEGYVQERTISFKDDGTYKTRAEVKFEGDTLVNRIELKGIDFKEDGNILGHKLEYNFNS
HNVYITADKQKNGIKANFKIRHNVED**GSGKLGSG**SVQLADHYQQNTPIGDGPVLLPDNHYLSTQSVLSKDPNEKRDHMVLLEF
VTAAGITHGMAELYK**GSGKLGSG**IEGRGHHHHHH

CTC-445.2d negative control (calc.: 38,374; obs.: 38,377)
MPGESLPETGGENLYFQGLSVEIDLGKGDFREIRASEDAREAAEALAEAARAMKEALEILREIAEKLRDSSRASEAAKRIAKA
IRKAADAIAEAAKIAARAAKDGDAARNAENAARKAKEFAEEQAKLADMYAELAKNGDKSSVLEQLKTFADKAFHEMEDLFYQA
ALAVFEAAEAAAGGGGSGALGSGSGGSGGGSPGSVEIDLGKGDFREIRASEDAREAAEALAEAARAMKEALEILREIAEKLRD
SSRASEAAKRIAKAIRKAADAIAEAAKIAARAAKDGDAARNAENAARKAKEFAEEQAKLADMYAELAKNGDKSSVLEQLKTFA
DKAFHEMEDLFYQAALAVFEAAEAAAGGSGWGIEGRHHHHHH

CTC-445.2d with KL-tag (calc.: 38,431; obs.: 38,434)
MPGESLPETGGENLYFQGLSVEIDLGKGDFREIRASEDAREAAEALAEAARAMKEALEILREIAEKLRDSSRASEAAKRIAKA
IRKAADAIAEAAKIAARAAKDGDAARNAENAARKAKEFAEEQAKLADMYAELAKNGDKSSVLEQLKTFADKAFHEMEDLFYQA
ALAVFEAAEAAAGGG**GSGKLGSG**SGGSGGGSPGSVEIDLGKGDFREIRASEDAREAAEALAEAARAMKEALEILREIAEKLRD
SSRASEAAKRIAKAIRKAADAIAEAAKIAARAAKDGDAARNAENAARKAKEFAEEQAKLADMYAELAKNGDKSSVLEQLKTFA
DKAFHEMEDLFYQAALAVFEAAEAAAGGSGWGIEGRHHHHHH

CTC-445.2d with KL-tag and C-terminal NGLH (calc.: 40,108; obs.: 40,110; no N-terminal label)
MPGESLPETGGENLYFQGLSVEIDLGKGDFREIRASEDAREAAEALAEAARAMKEALEILREIAEKLRDSSRASEAAKRIAKA
IRKAADAIAEAAKIAARAAKDGDAARNAENAARKAKEFAEEQAKLADMYAELAKNGDKSSVLEQLKTFADKAFHEMEDLFYQA
ALAVFEAAEAAAGGG**GSGKLGSG**SGGSGGGSPGSVEIDLGKGDFREIRASEDAREAAEALAEAARAMKEALEILREIAEKLRD
SSRASEAAKRIAKAIRKAADAIAEAAKIAARAAKDGDAARNAENAARKAKEFAEEQAKLADMYAELAKNGDKSSVLEQLKTFA
DKAFHEMEDLFYQAALAVFEAAEAAAGGSGWGSGSNGLHGIEGRHHHHHH

VHH_{6e}-VHH_{MHCII} negative control (calc.: 28,763; obs.: 28,765)
MPSSLPETGGQVQLQESGGGLVQPGGSLRLSCAASGFVFENSAMAWYRQAPGKERELIAVIGTTFIKLAESVKGRFTISRDNA
KSTVYLQMNNLKPEDTAVYYCSKSGAYWGQGTQVTVSSGGSGALGSGGSGAPQVQLQESGGGLVQAGDSLRLSCAASGRTFSR
GVMGWFRRAPGKEREFVAIFSGSSWSGRSTYYSDSVKGRFTISRDNAKNTVYLQMNGLKPEDTAVYYCAAGYPEAYSAYGRES
TYDYWGQGTQVTVSSGENLYFQGHHHHHH

VHH_{6e}-VHH_{MHCII} with KL-tag (calc.: 28,820; obs.: 28,822)
MPSSLPETGGQVQLQESGGGLVQPGGSLRLSCAASGFVFENSAMAWYRQAPGKERELIAVIGTTFIKLAESVKGRFTISRDNA
KSTVYLQMNNLKPEDTAVYYCSKSGAYWGQGTQVTVSSG**GSGKLGSG**GSGAPQVQLQESGGGLVQAGDSLRLSCAASGRTFSR
GVMGWFRRAPGKEREFVAIFSGSSWSGRSTYYSDSVKGRFTISRDNAKNTVYLQMNGLKPEDTAVYYCAAGYPEAYSAYGRES
TYDYWGQGTQVTVSSGENLYFQGHHHHHH

4. Supplementary figures

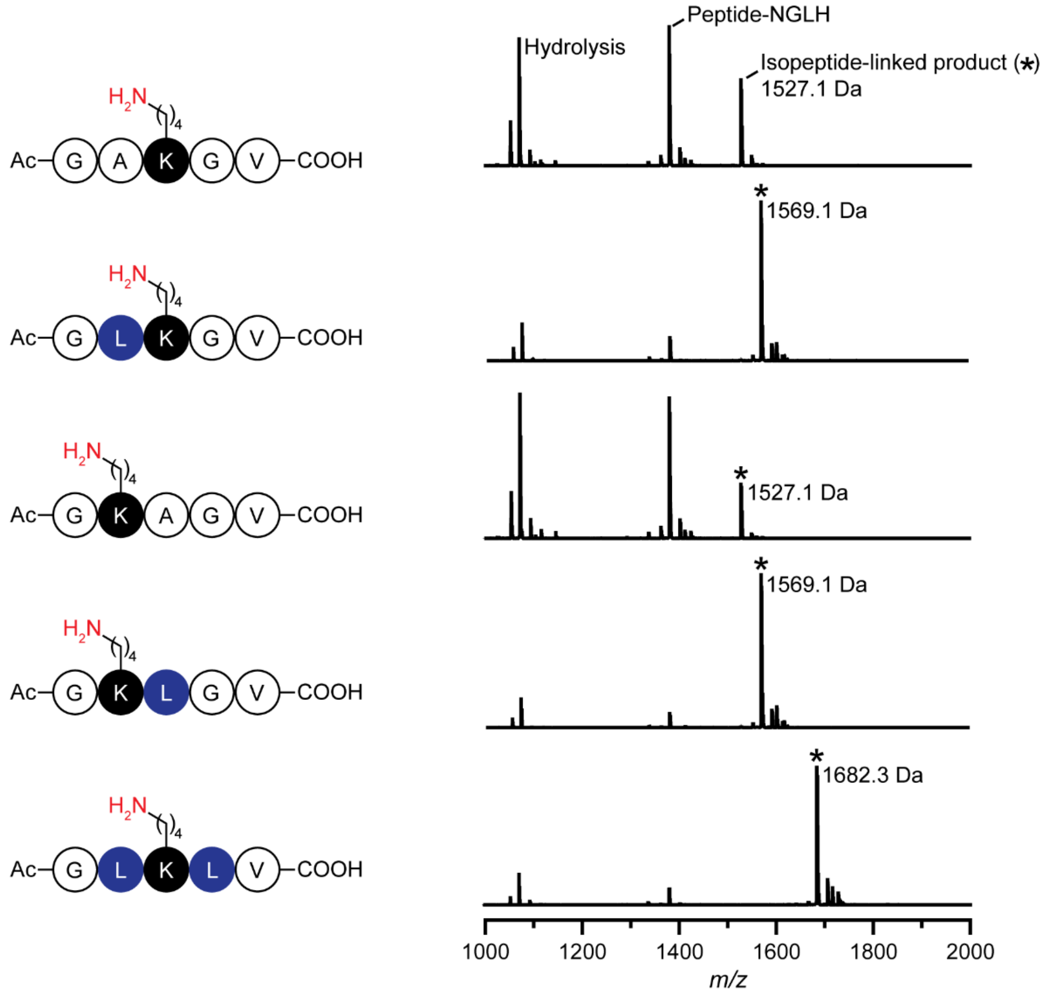

Supplementary Figure 1. MALDI-TOF MS analysis of the reactions shown in Fig. 2b. Ligation of a model NGLH-containing acyl donor peptide (Ac-RWRGWRNGLH, 0.1 mM) to Lys-containing acyl acceptor peptides (0.5 mM) as catalysed by 1 µM *Oa*AEP1 in HEPES buffer containing 0.2 mM $NiSO_4$, pH 8.5, after 1.5 h at 25°C.

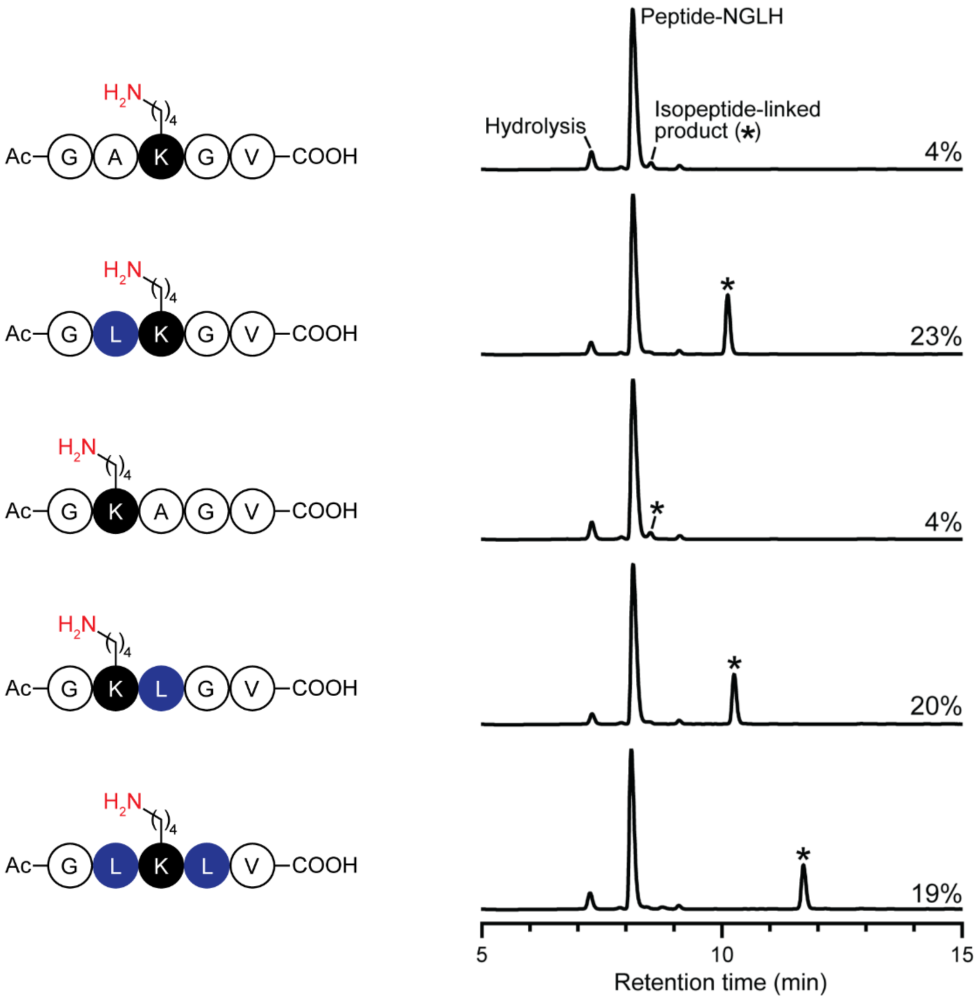

Supplementary Figure 2. RP-HPLC analysis of reactions as shown in Fig. 2b but without Ni^{2+} addition. Ligation of a model NGLH-containing acyl donor peptide (Ac-RWRGWRNGLH, 0.1 mM) to Lys-containing acyl acceptor peptides (0.5 mM) as catalysed by 1 µM *Oa*AEP1 in HEPES buffer, pH 8.5, after 1.5 h at 25°C.

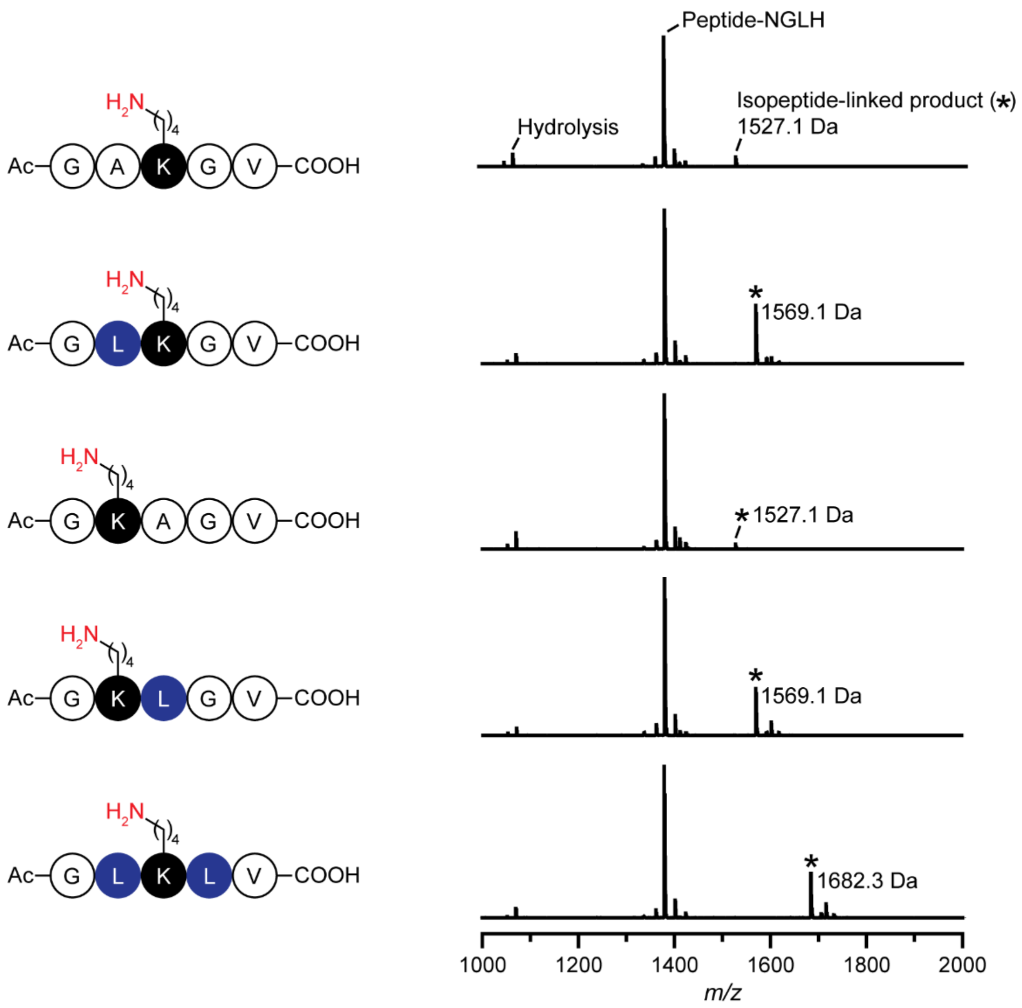

Supplementary Figure 3. MALDI-TOF MS analysis of the reactions shown in Supplementary Fig. 2. Ligation of a model NGLH-containing acyl donor peptide (Ac-RWRGWRNGLH, 0.1 mM) to Lys-containing acyl acceptor peptides (0.5 mM) as catalysed by 1 µM *Oa*AEP1 in HEPES buffer, pH 8.5, after 1.5 h at 25°C.

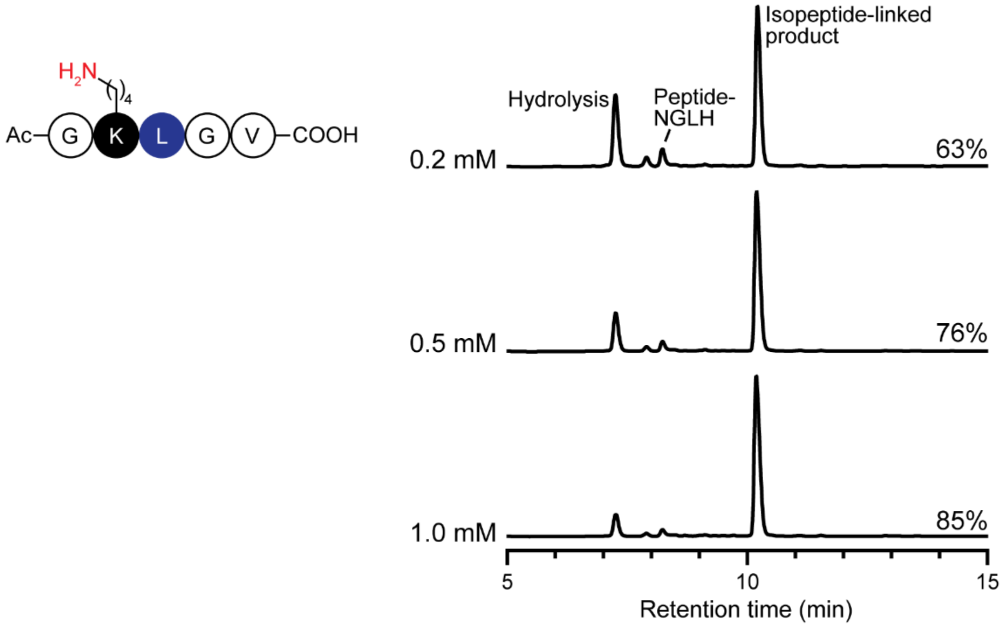

Supplementary Figure 4. RP-HPLC analysis of reactions at the indicated concentrations of the shown KL-containing peptide (Ac-GKLGV) carried out as in Fig. 2b. Ligation of a model NGLH-containing acyl donor peptide (Ac-RWRGWRNGLH, 0.1 mM) to Lys-containing peptides (0.2-1 mM) as catalysed by 1 µM *Oa*AEP1 in HEPES buffer containing 0.2 mM $NiSO_4$, pH 8.5, after 1.5 h at 25°C.

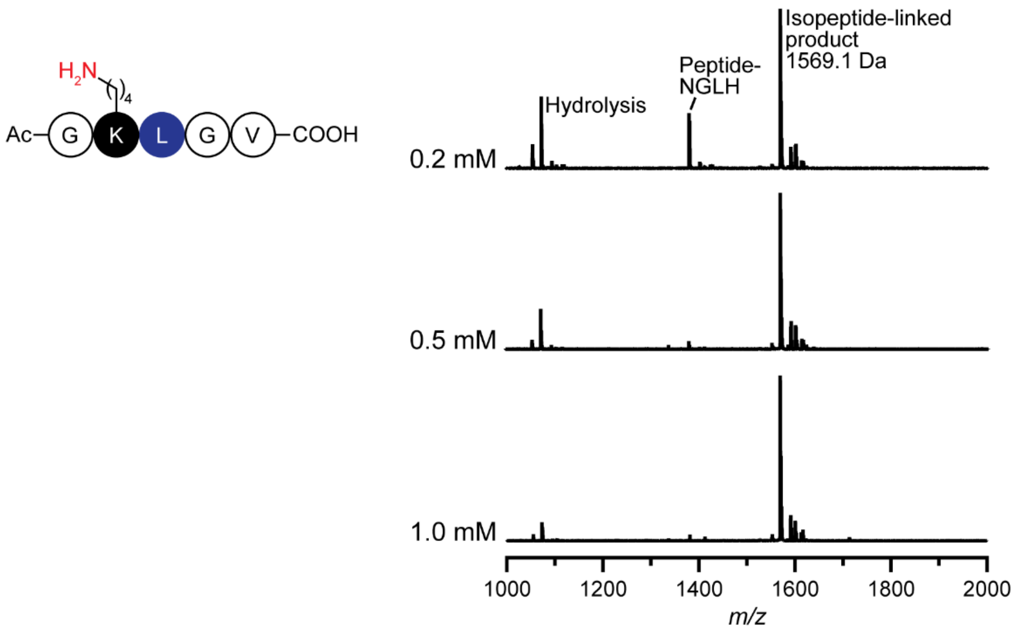

Supplementary Figure 5. MALDI-TOF MS analysis of the reactions shown in Supplementary Fig. 4. Ligation of a model NGLH-containing acyl donor peptide (Ac-RWRGWRNGLH, 0.1 mM) to Lys-containing peptides (0.2-1 mM) as catalysed by 1 µM *Oa*AEP1 in HEPES buffer containing 0.2 mM $NiSO_4$, pH 8.5, after 1.5 h at 25°C.

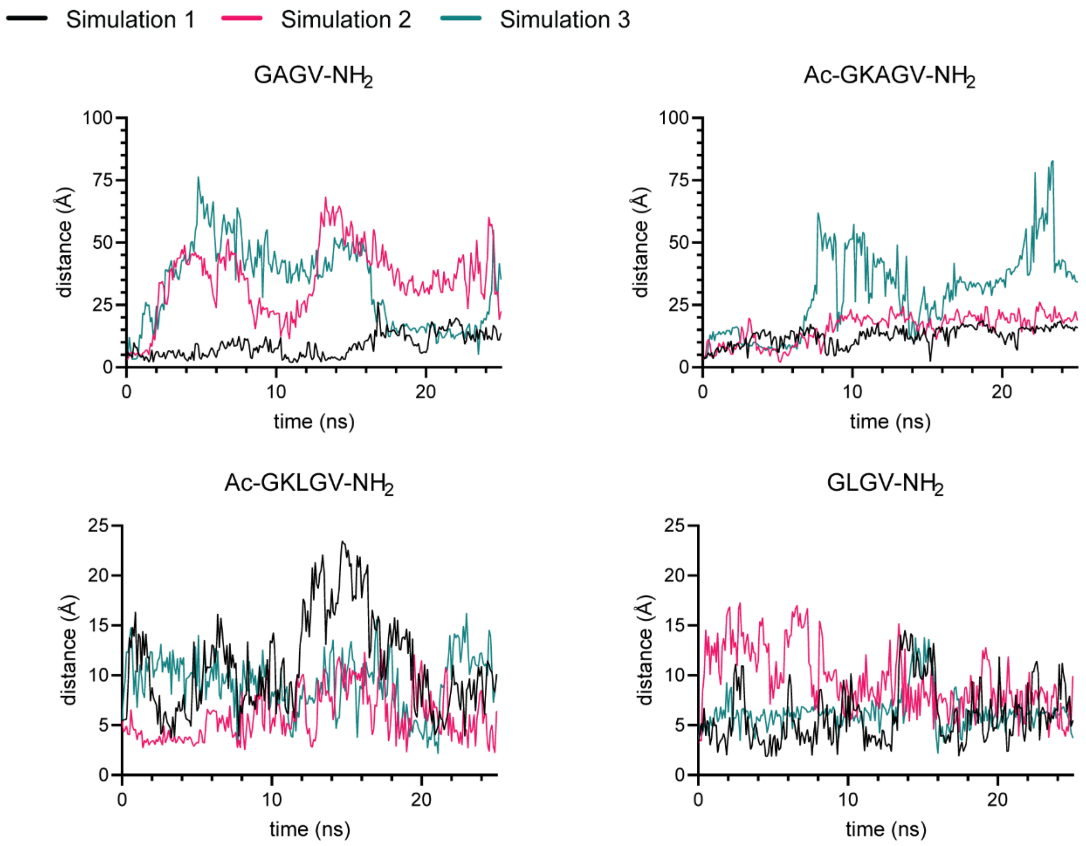

Supplementary Figure 6. Analysis of canonical substrates and Lys-containing mimetics via molecular dynamics simulations. We generated homology models of an *Oa*AEP1 acyl-enzyme intermediate (using Ac-RN as a model acyl donor) in complex with an acyl acceptor peptide substrate, either **GL**GV-NH_2, Ac-G**KL**GV-NH_2, **G**AGV-NH_2 or Ac-G**K**AGV-NH_2. The distances of the indicated simulated acyl acceptor amines (α or ε) to the thioester carbonyl carbon of Asn in the acyl-enzyme intermediate were plotted for three simulations.

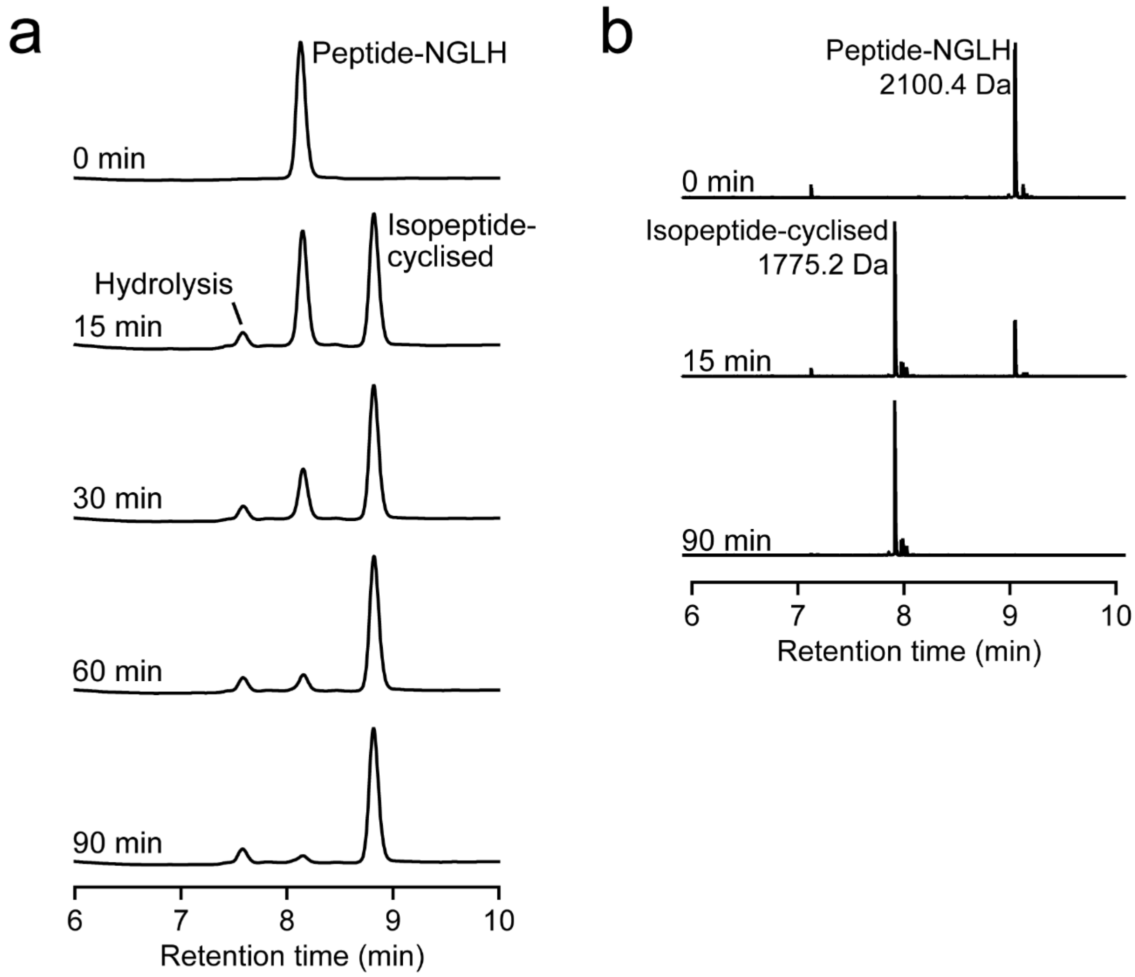

Supplementary Figure 7. a, Representative RP-HPLC (214 nm) and, **b,** MALDI-TOF MS analysis of the time course reactions shown in Fig. 3b. The reactions contained 20 µM peptide (Ac-GCGS**KL**GSCGHfRWGSNGLH) and 200 nM *Oa*AEP1 and were run in 100 mM HEPES buffer, pH 7.5, at 25°C for the indicated durations.

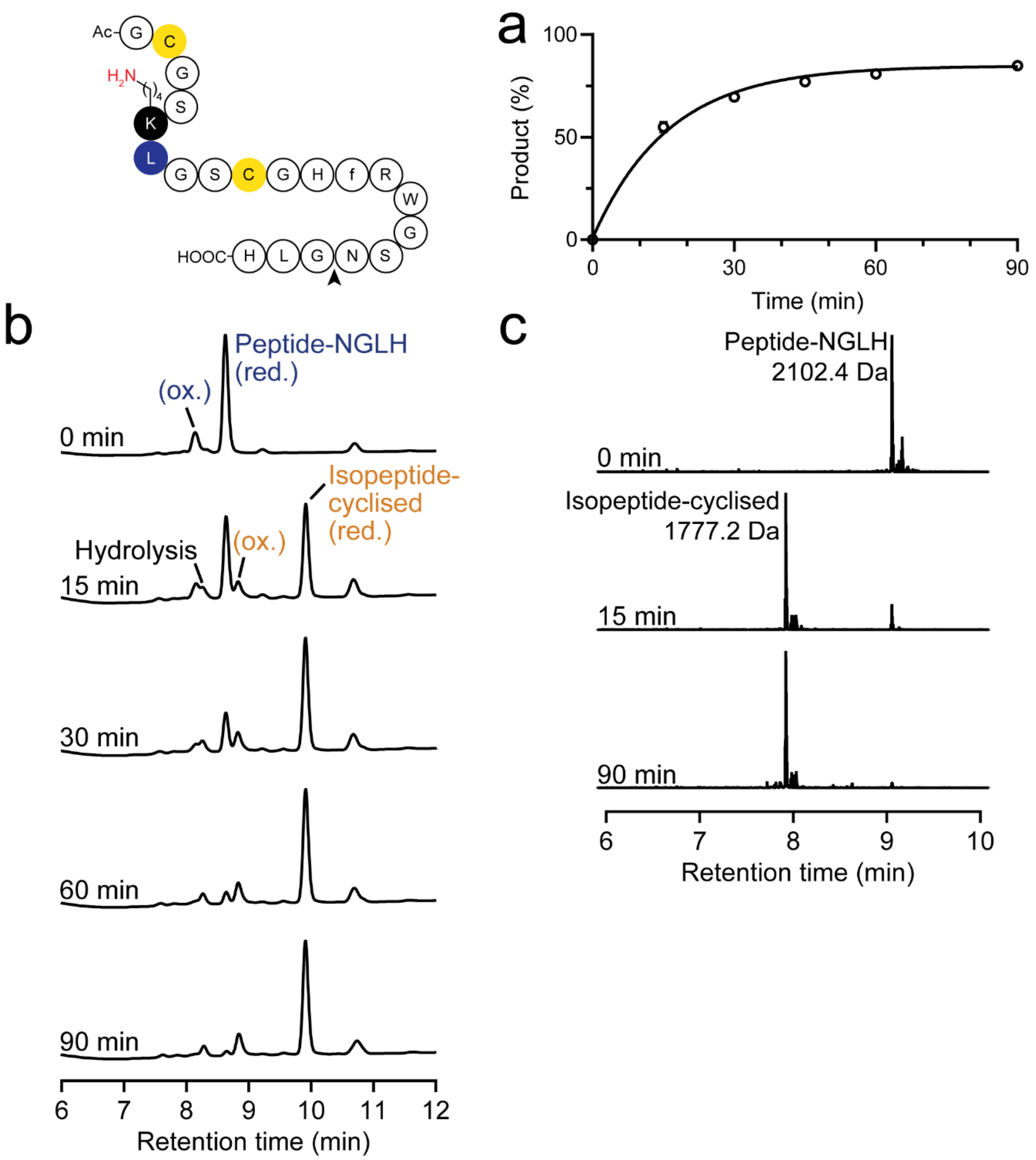

Supplementary Figure 8. Analysis of reactions as in Fig. 3b but carried out on a reduced substrate. A small amount of oxidised substrate was still present (as labelled). **a,** Isopeptide-cyclised product formation of the reduced model peptide substrate shown in in the upper left, quantified by RP-HPLC (relative peak integrals; *n* = 3 independent experiments; mean ± SD; error bars are too small to see at some points). **b,** Representative RP-HPLC spectra (214 nm) of the time course reactions shown in (**a**). **c,** MALDI-TOF MS analysis of select timepoints from (**b**). The reactions contained 20 µM reduced peptide (Ac-GCGS**KL**GSCGHfRWGSNGLH) and 200 nM *Oa*AEP1 and were run in 100 mM HEPES buffer, pH 7.5, at 25°C for the indicated durations.

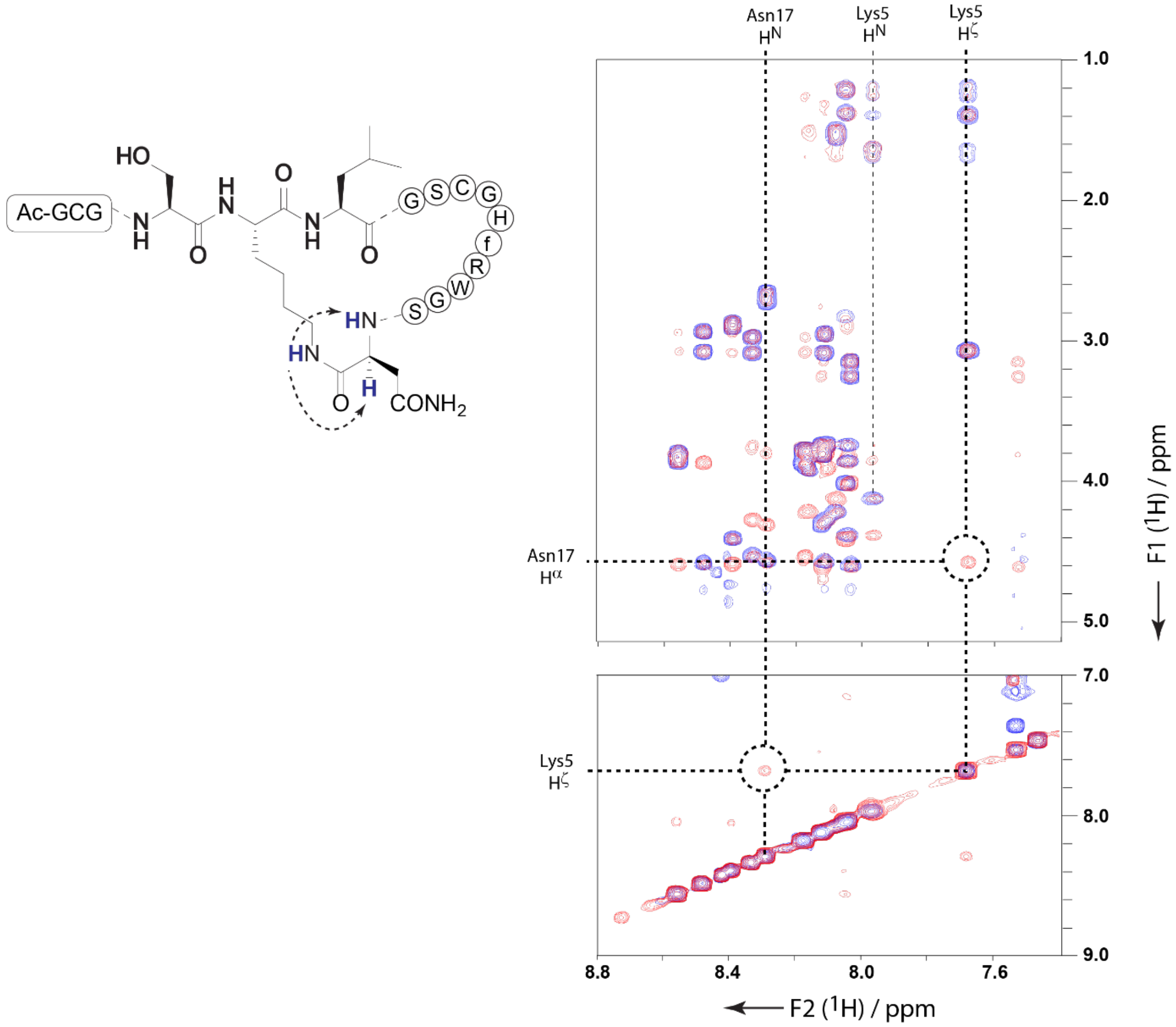

Supplementary Figure 9. NMR analysis of isopeptide bond-cyclised model peptide substrate (Ac-GCGSKLGSCGHfRWGSN). Superimposed TOCSY (blue contours) and NOESY (red contours) spectra of the peptide fingerprint region. Nuclear Overhauser effect cross peaks between the Lys5 H^{ζ} proton and the Asn17 H^{α} and H^{N} protons (circled) support the anticipated structure. The two cysteine residues in this peptide have been oxidised to form a disulfide bond. Spectra were acquired on a 600 MHz spectrometer in H_2O/D_2O (9:1) at 298 K.

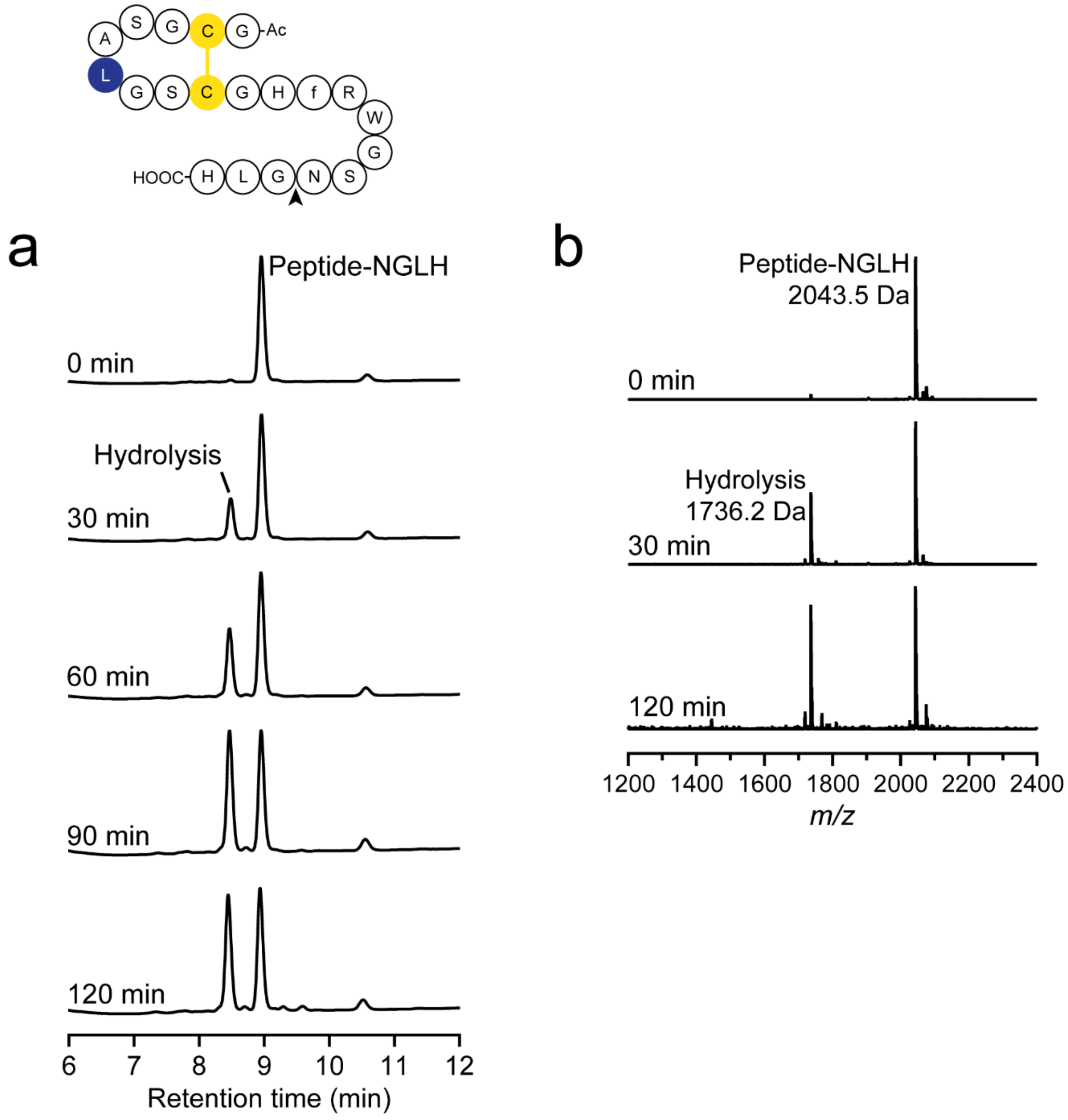

Supplementary Figure 10. Analysis of reactions carried out as in Fig. 3b on a substrate lacking an internal Lys (as depicted above; Ac-GCGSALGSCGHfRWGSNGLH). **a,** RP-HPLC (214 nm). **b,** MALDI-TOF MS. The reactions contained 20 µM peptide and 200 nM *Oa*AEP1 and were run in 100 mM HEPES buffer, pH 7.5, at 25°C for the indicated durations.

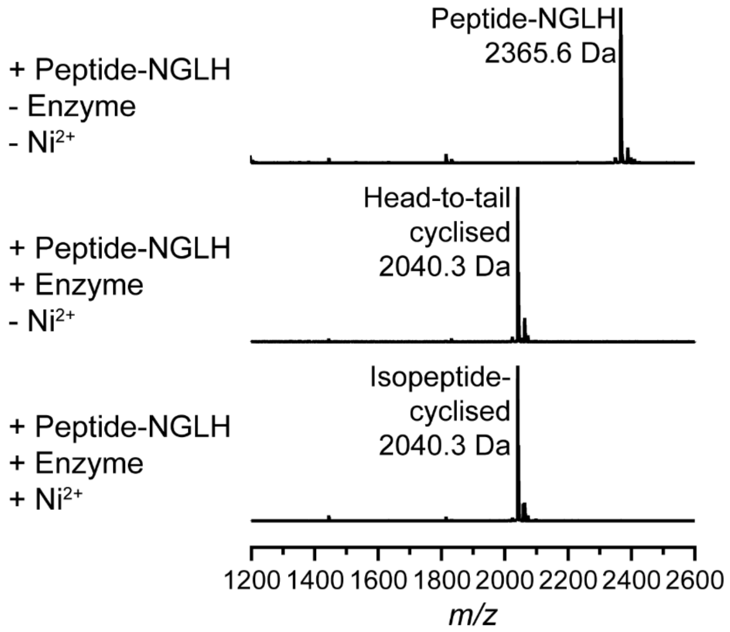

Supplementary Figure 11. MALDI-TOF MS analysis of the reactions shown in Fig. 3d. The reactions contained 20 µM peptide (GLHGCGS**KL**GSCGHfRWGSNGLH) and 200 nM *Oa*AEP1, with or without 100 µM $NiSO_4$, and were run in 100 mM HEPES buffer, pH 7.5, at 25°C for 3 h.

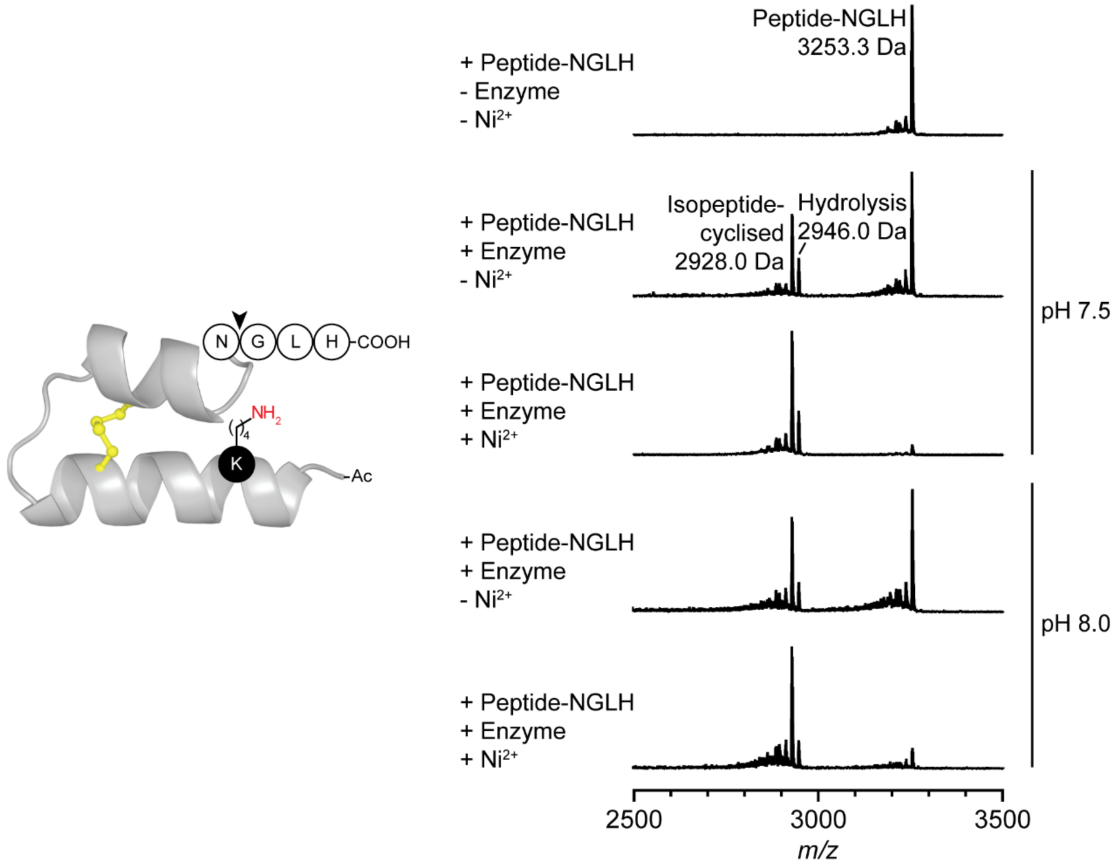

Supplementary Figure 12. MALDI-TOF MS analysis of reactions as shown in Fig. 3e but on a substrate lacking a Leu residue adjacent to the Lys (Ac-GSTTA**K**NIYNTCRFGGGSRTLCARLSGNGLH). Hydrolysis is partially suppressed by shifting reaction pH from 7.5 to 8.0. The reactions contained 20 µM peptide and 400 nM *Oa*AEP1, with or without 40 µM $NiSO_4$, and were run in 100 mM HEPES buffer, pH 7.5 or 8.0, at 25°C for 3 h.

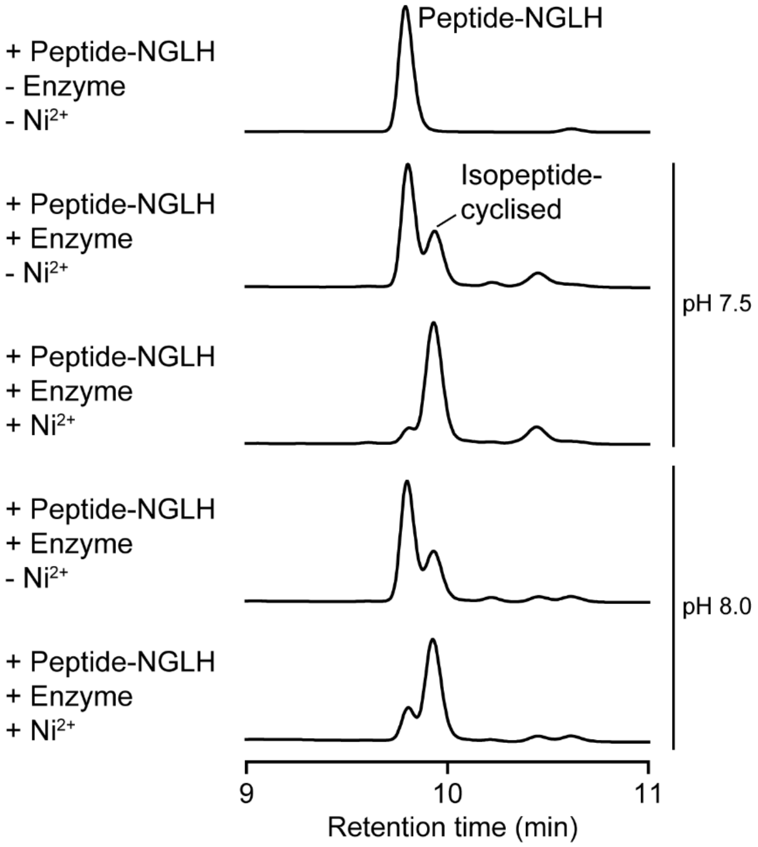

Supplementary Figure 13. RP-HPLC (214 nm) analysis of the reactions shown in Supplementary Fig. 12. The reactions contained 20 µM peptide (Ac-GSTTA**K**NIYNTCRFGGGSRTLCARLSG<u>NGL</u>H) and 400 nM *Oa*AEP1, with or without 40 µM $NiSO_4$, and were run in 100 mM HEPES buffer, pH 7.5 or 8.0, at 25°C for 3 h.

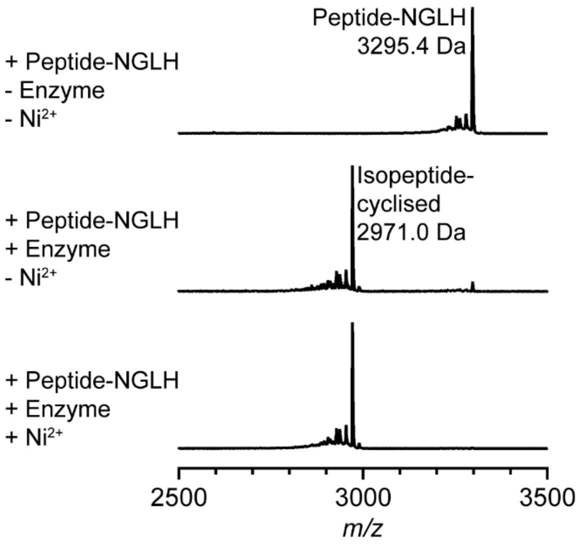

Supplementary Figure 14. MALDI-TOF MS analysis of the reaction shown in Fig. 3e (lower and upper panels are also shown in Fig. 3e) in addition to a control reaction without Ni^{2+}. The reactions contained 20 µM peptide (Ac-GSTT**LK**NIYNTCRFGGGSRTLCARLSGNGLH) and 400 nM *Oa*AEP1, with or without 40 µM $NiSO_4$, and were run in 100 mM HEPES buffer, pH 7.5, at 25°C for 3 h.

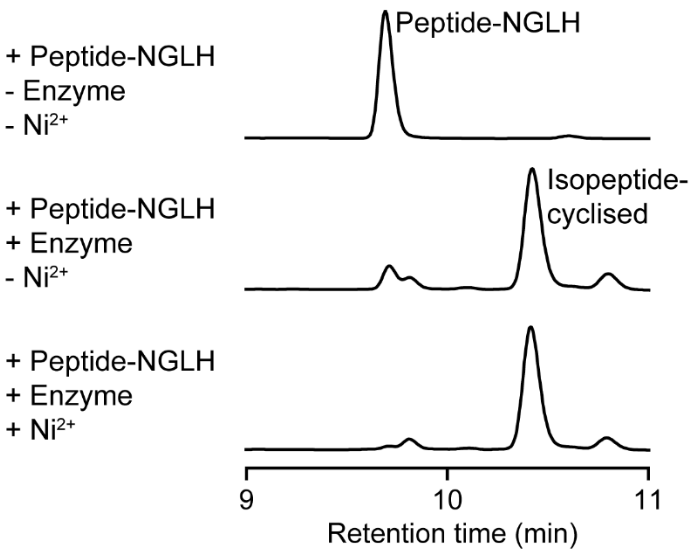

Supplementary Figure 15. RP-HPLC (214 nm) analysis of the reactions shown in Supplementary Fig. 14. The reactions contained 20 µM peptide (Ac-GSTT**LK**NIYNTCRFGGGSRTLCARLSGNGLH) and 400 nM *Oa*AEP1, with or without 40 µM $NiSO_4$, and were run in 100 mM HEPES buffer, pH 7.5, at 25°C for 3 h.

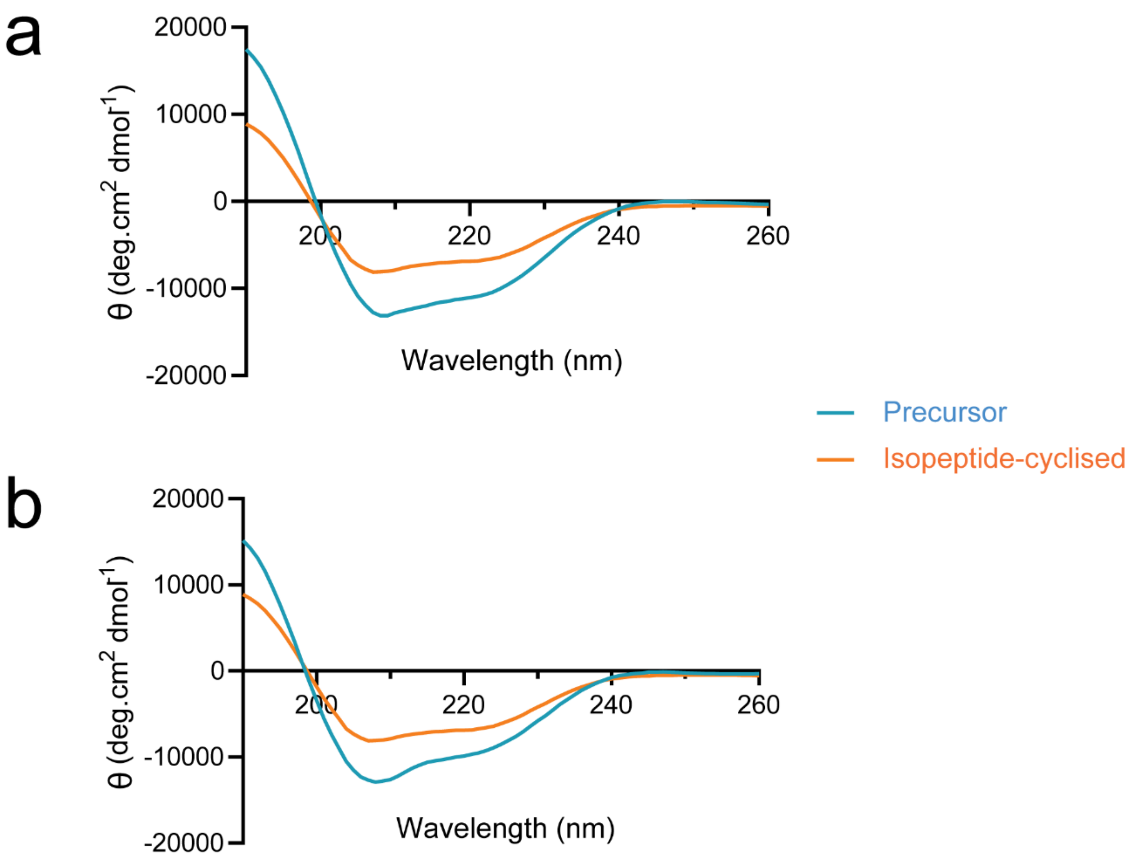

Supplementary Figure 16. Circular dichroism spectra of truncated stinging tree-derived crambin-like peptides. Minima between 205 and 222 nm indicate a helical conformation for both the precursor and isopeptide-cyclised product. **a,** overall helicity 35 and 22% for the precursor and isopeptide-cyclised samples, respectively (Ac-GSTTA**K**NIYNTCRFGGGSRTLCARLSGNGLH). **b,** overall helicity 32 and 22% for the precursor and isopeptide-cyclised samples, respectively (Ac-GSTT**LK**NIYNTCRFGGGSRTLCARLSGNGLH).

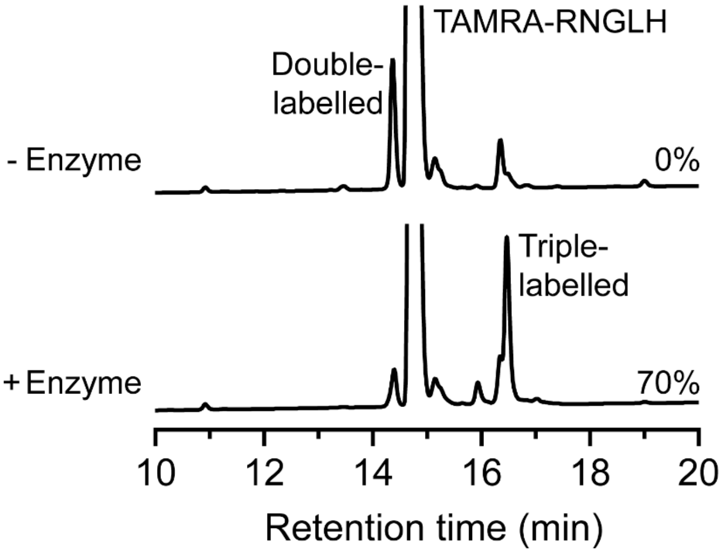

Supplementary Figure 17. RP-HPLC (214 nm) analysis of the attachment of TAMRA-RN to GLH**KL**GRL-Eda that was double-labelled with biotin-RN in the previous step, as shown in Fig. 4c. In the upper chromatogram the TAMRA-RNGLH peak extends beyond the height of the y-axis – the scale for the upper and lower panels is the same. Conversion (%) to the triple labelled product was calculated based on relative peak heights.

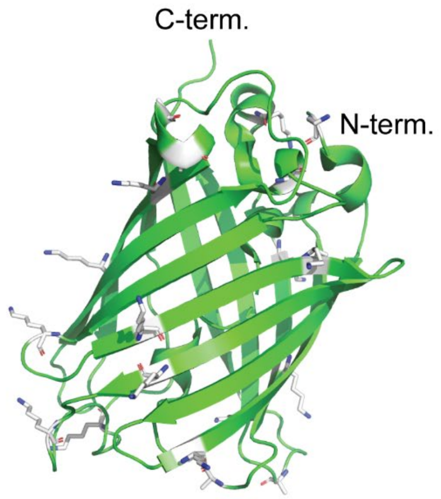

Supplementary Figure 18. Structure of sfGFP (PDB: 2B3P) with Lys residues highlighted in white. Lys238 (the most C-terminal Lys) is not resolved in this structure.

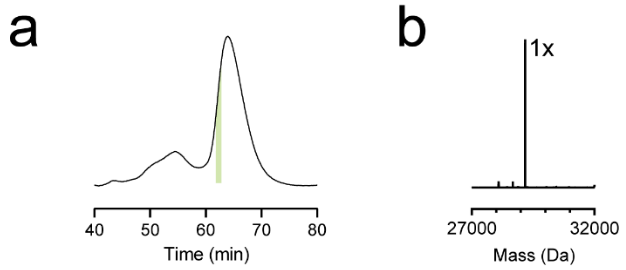

Supplementary Figure 19. a, Size exclusion chromatography on a Superdex 75pg HiLoad 16/600 column of the crude labelling reaction of sfGFP with a C-terminal KL-tag labelled with biotin-RN. The labelling reaction was set as described in Fig. 5b but for 4 h. The column was equilibrated with 50 mM sodium phosphate buffer containing 150 mM sodium chloride, pH 7. The analysed fraction is highlighted in green. **b,** Reconstructed ESI-MS spectrum of the fraction highlighted in (**a**).

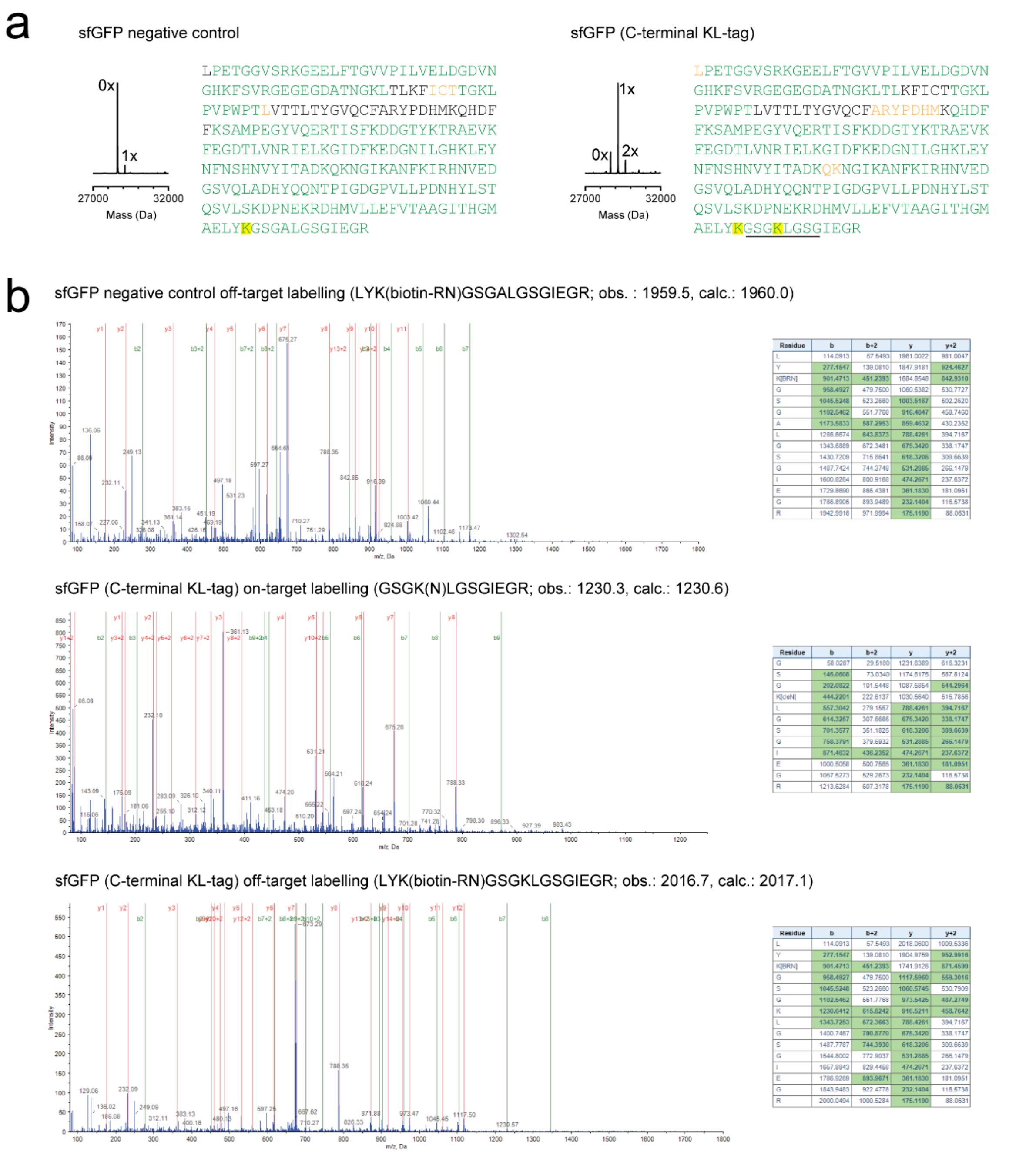

Supplementary Figure 20. a, Reconstructed ESI-MS spectra of C-terminally KL-tagged sfGFP or the negative control substrate labelled with biotin-RN as described in Fig. 5b. The coverage from the MS/MS analysis of each reaction, as determined by ProteinPilot, is shown in the sequence adjacent where green indicates that a peptide was identified with at least 95% confidence and yellow indicates at least 50% confidence. The KL-tag is underlined and on- or off-target labelling sites are indicated by a yellow highlight. **b,** MS/MS spectra that span the on- or off-target labelling sites for the KL-tagged or control substrates as assigned by ProteinPilot. The highlighted y and b ion masses in the adjacent tables indicate which ions were identified in each spectrum. If the spectrum derived from a tryptic digest, the modification on the Lys residue is only an Asn as the rest of the biotin-RN peptide was cleaved off.

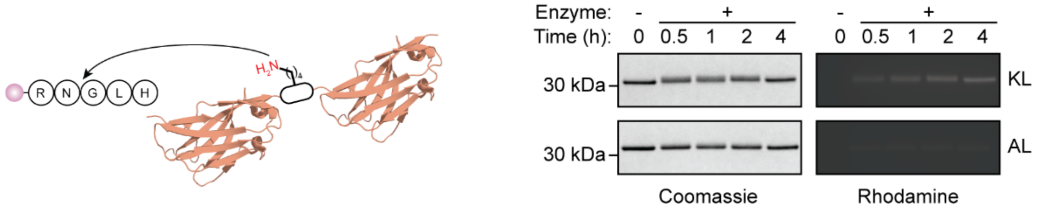

Supplementary Figure 21. SDS-PAGE analysis of the indicated reactions shown in Extended Data Fig. 6. Reactions were run in 100 mM HEPES buffer, pH 8, containing 50 µM protein, 1 mM TAMRA-RNGLH, 1 mM $NiSO_4$, and 1 µM *Oa*AEP1 for the indicated timepoints at 25°C. Rhodamine fluorescence was imaged for identical exposure times and Coomassie staining was conducted using InstantBlue. The rhodamine gel contrasts were adjusted to be similar to Fig. 5d which was modified in the editorial process. Unprocessed gels are shown on page S37.

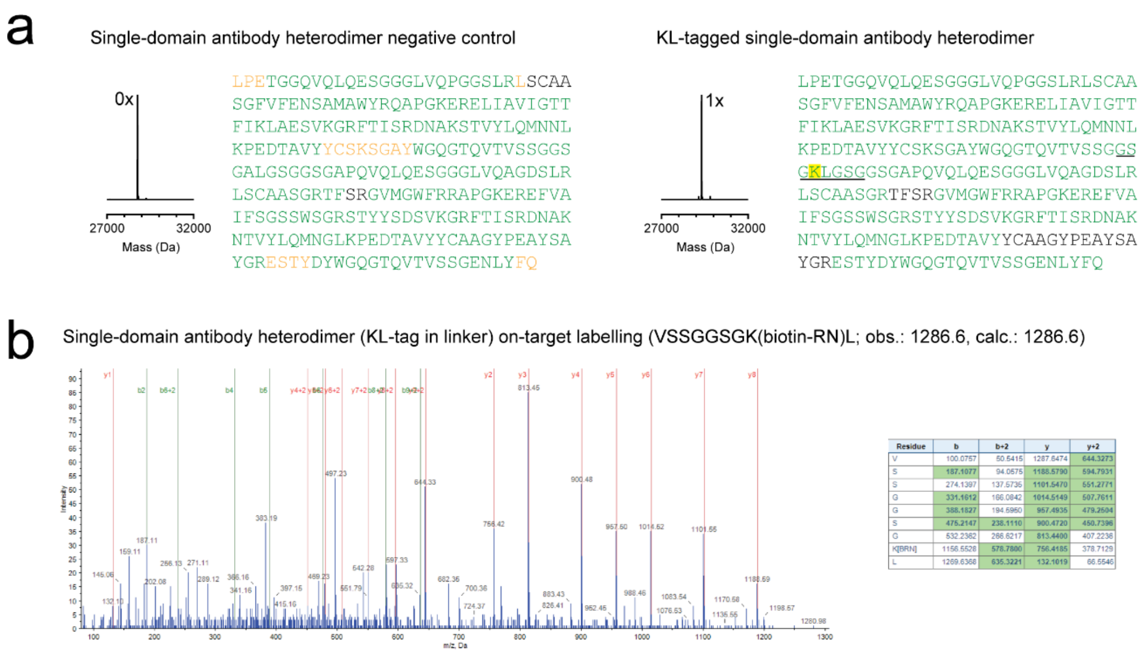

Supplementary Figure 22. a, Reconstructed ESI-MS spectra of the KL-tagged single-domain antibody heterodimer or the negative control substrate labelled with biotin-RN as described in Fig. 5c. The coverage from the MS/MS analysis of each reaction, as determined by ProteinPilot, is shown in the sequence adjacent where green indicates that a peptide was identified with at least 95% confidence and yellow indicates at least 50% confidence. The KL-tag is underlined and the on-target labelling site is indicated by a yellow highlight. **b,** MS/MS spectrum that spans the on-target labelling site for the KL-tagged substrate as assigned by ProteinPilot. The highlighted y and b ion masses in the adjacent table indicate which ions were identified in the spectrum.

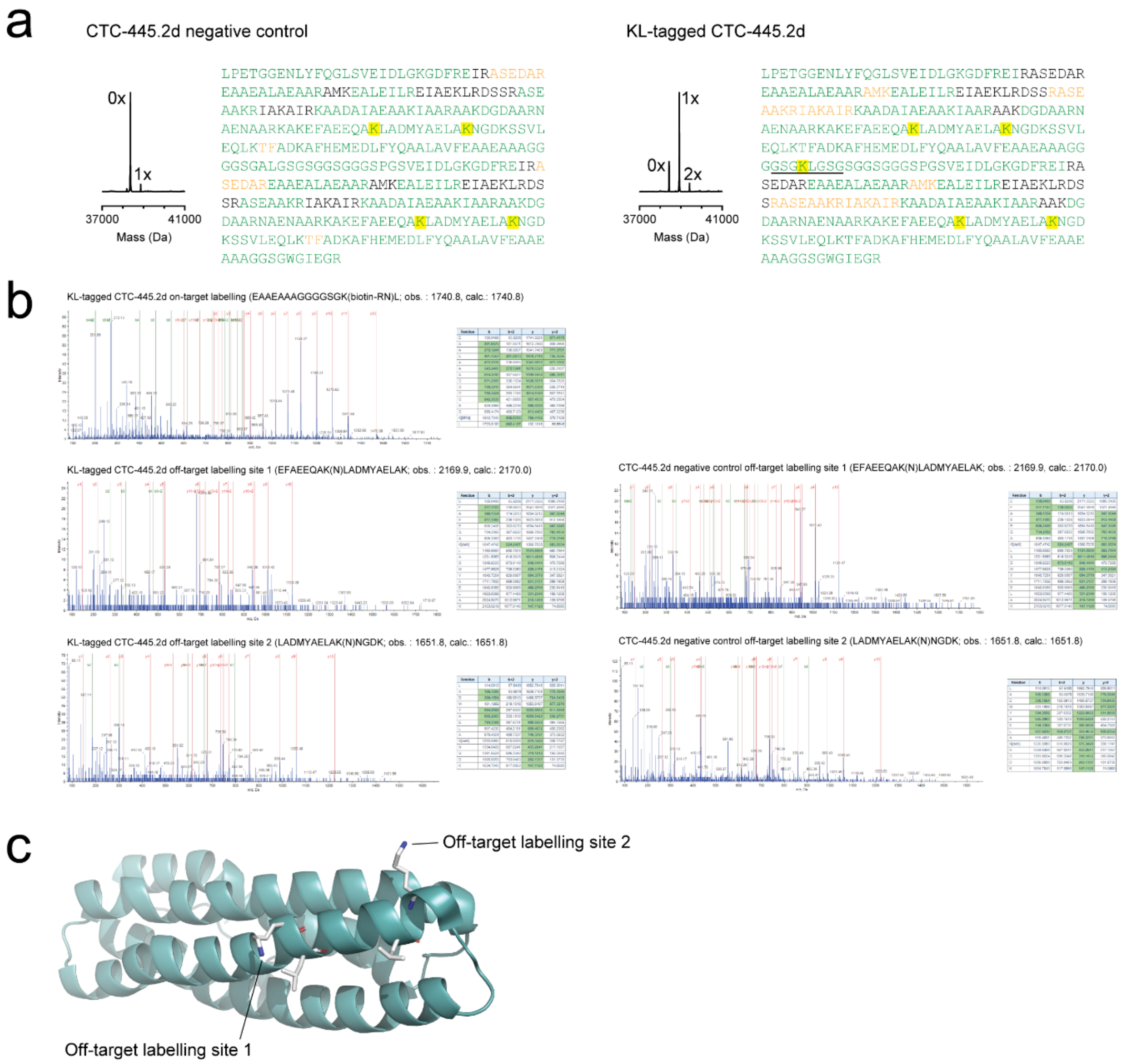

Supplementary Figure 23. a, Reconstructed ESI-MS spectra of KL-tagged CTC-445.2d or the negative control substrate labelled with biotin-RN as described in Fig. 5d. The coverage from the MS/MS analysis of each reaction, as determined by ProteinPilot, is shown in the sequence adjacent where green indicates that a peptide was identified with at least 95% confidence and yellow indicates at least 50% confidence. The KL-tag is underlined and on- or off-target labelling sites are indicated by a yellow highlight. Since this substrate is a homodimer, the two identified off-target labelling sites occur in each monomer. **b,** MS/MS spectra that span the on- or off-target labelling sites for the KL-tagged or control substrates as assigned by ProteinPilot. The highlighted y and b ion masses in the adjacent tables indicate which ions were identified in each spectrum. If the spectrum derived from a tryptic digest, the modification on the Lys residue is only an Asn as the rest of the biotin-RN peptide was cleaved off. **c,** Structure of CTC-445 (monomer; PDB: 7KL9) with the off-target labelling Lys residues and adjacent or structurally proximal Leu residues shown in white.

5. Unprocessed gel images

Shown in Supplementary Fig. 21

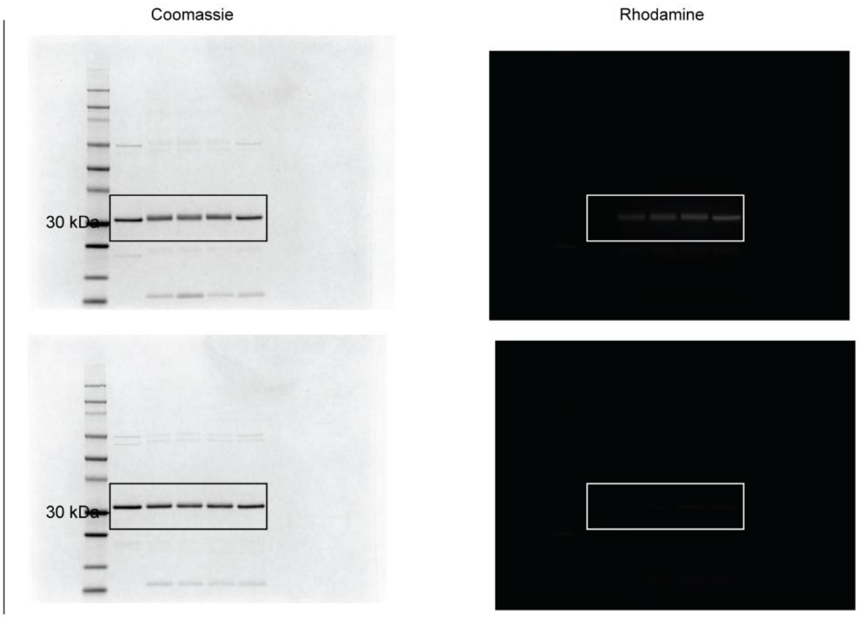

6. Supporting references

1 Rehm, F. B. H., Tyler, T. J., Yap, K., Durek, T. & Craik, D. J. Improved Asparaginyl-Ligase-Catalyzed Transpeptidation via Selective Nucleophile Quenching. *Angew Chem Int Ed Engl* **60**, 4004-4008, doi:10.1002/anie.202013584 (2021).

2 Rehm, F. B. H. *et al.* Enzymatic C-Terminal Protein Engineering with Amines. *J Am Chem Soc* **143**, 19498-19504, doi:10.1021/jacs.1c08976 (2021).

3 Rehm, F. B. H., Tyler, T. J., de Veer, S. J., Craik, D. J. & Durek, T. Enzymatic C-to-C Protein Ligation. *Angew Chem Int Ed Engl* **61**, e202116672, doi:10.1002/anie.202116672 (2022).
