# Supplementary figures and images for: Repurposing a plant peptide cyclase for targeted lysine acylation
Source: Nat Chem. 2024 May 24;16(9):1481–9. doi: 10.1038/s41557-024-01520-1 (PMC11374674; doi:10.1038/s41557-024-01520-1)

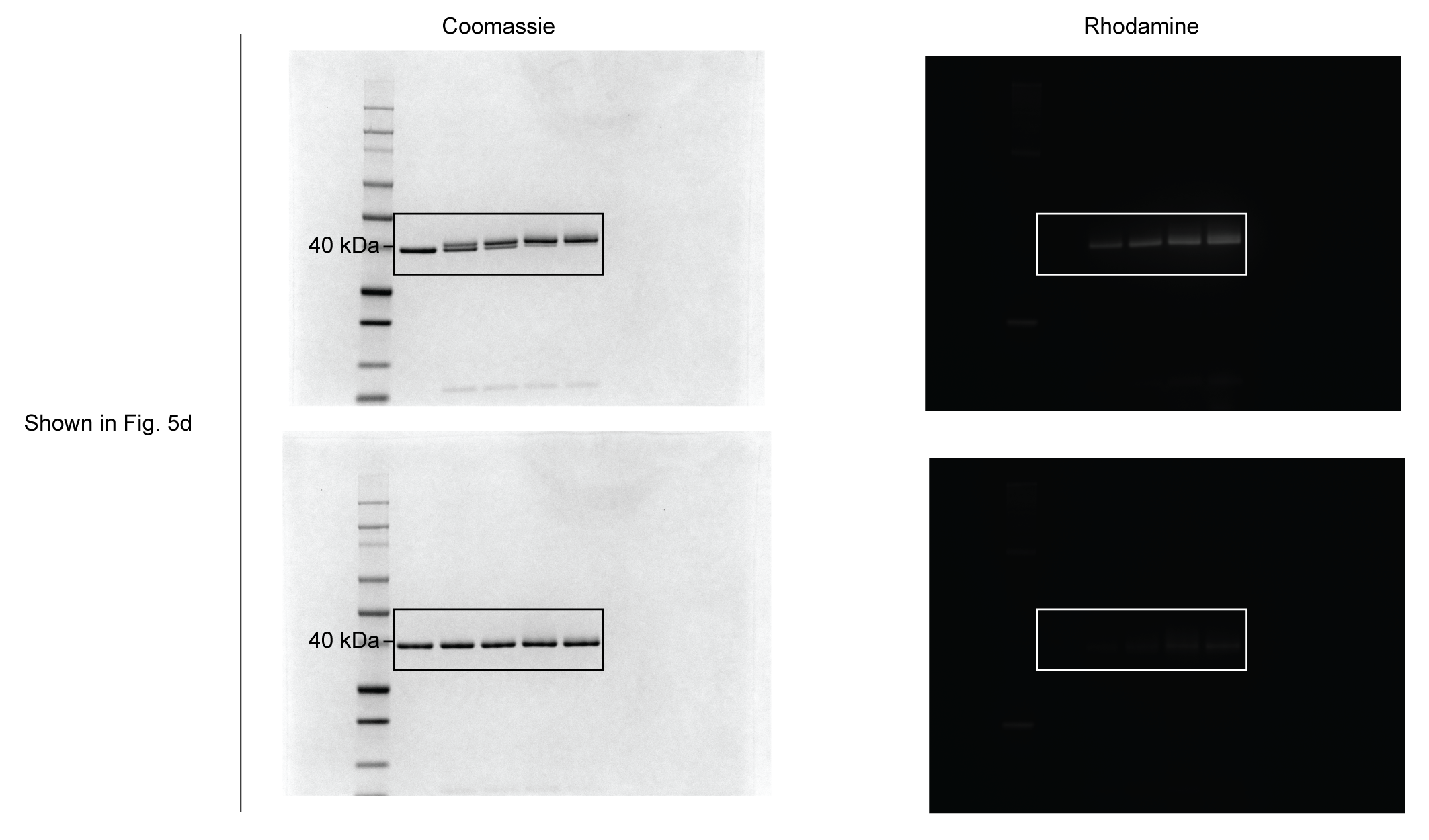

Supplement: Supplementary file 5 — Unprocessed gels. [file 41557_2024_1520_MOESM5_ESM.tif]
